# Regulation of Somatosensory Temporal Discrimination Threshold Through Motor Training: An EEG and Kinematics Study

**DOI:** 10.1111/cns.70564

**Published:** 2025-08-26

**Authors:** Jinyan Zhang, Wangjun Zou, Binbin Gao, Jinglong Wu, Zhilin Zhang, Jian Zhang, Luyao Wang, Tianyi Yan

**Affiliations:** ^1^ School of Mechatronical Engineering Beijing Institute of Technology Beijing China; ^2^ School of Medical Technology Beijing Institute of Technology Beijing China; ^3^ School of Life Science Beijing Institute of Technology Beijing China; ^4^ Research Center for Medical Artificial Intelligence Shenzhen Institute of Advanced Technology, Chinese Academy of Sciences Shenzhen Guangdong China; ^5^ Department of Psychiatry, Graduate School of Medicine Kyoto University Kyoto Japan; ^6^ Institute of Biomedical Engineering, School of Life Sciences Shanghai University Shanghai China

**Keywords:** EEG, kinematic analysis, motor training, sensorimotor integration, SEP, STDT

## Abstract

**Aims:**

Motor training enhances somatosensory temporal discrimination threshold (STDT), but the distinct neural mechanisms underlying actual execution versus motor imagery remain unclear. This study aimed to compare the effects of ball‐rotation training (BRT; actual execution) and visual‐guided imagery (VGI; motor imagery) on STDT, kinematic performance, and neurophysiological plasticity in healthy adults.

**Methods:**

Forty‐eight right‐handed participants were randomized into four groups: BRT (actual execution), VGI (motor imagery without movement), tactile control (simple gripping), and no‐intervention control. Over seven days, participants underwent pre‐/post‐training assessments including kinematic analysis, STDT measurement, power spectral analysis and somatosensory‐evoked potentials (SEPs).

**Results:**

BRT significantly enhanced motor performance (83% score increase vs. 21% in controls, *p* < 0.001) and movement speed (37% cycle time reduction vs. 12%–16% in others, *p* < 0.001), with partial transfer to the untrained hand. Both interventions reduced STDT but at distinct locations: BRT selectively improved index finger discrimination (64.02 ms → 43.75 ms, *p* = 0.007), while VGI enhanced palm sensitivity (73.43 ms → 61.13 ms, *p* = 0.003). Neurophysiologically, SEPs revealed increased spatial inhibition ratio (SIR) plasticity in both BRT and VGI (*p* < 0.001), correlating with STDT gains. EEG demonstrated BRT‐induced gamma‐band power increases in parietal regions and theta‐band elevations in prefrontal cortex, whereas VGI modulated delta‐band activity in ipsilateral parietal cortex.

**Conclusion:**

Actual execution (BRT) and motor imagery (VGI) enhance STDT through distinct neuroplastic mechanisms: BRT optimizes sensorimotor integration via parietal gamma/prefrontal theta oscillations, while VGI relies on ipsilateral parietal delta modulation. These findings underscore the role of cortical reorganization in motor learning and support tailored rehabilitation strategies for neurological disorders.

## Graphical abstract

Actual execution enhanced motor performance and reduced STDT at the index finger via parietal gamma and prefrontal theta plasticity, with increased SIR plasticity. Motor imagery (MI) improved palm STDT through ipsilateral parietal delta modulation, also enhancing SIR. Both paradigms induced distinct neural mechanisms linked to SEP plasticity and STDT improvements.

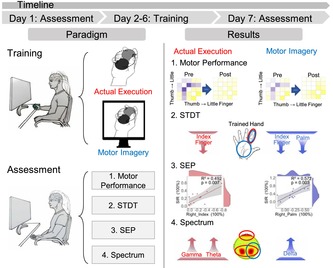

## 1 Introduction

The temporal precision of somatosensory processing is fundamental to motor control, allowing the nervous system to integrate sensory feedback with movements at millisecond‐level accuracy [1, 2]. This capability, quantified by the somatosensory temporal discrimination threshold (STDT), which is the minimum interval required to perceive two tactile stimuli as distinct [3], is regulated by a network involving the dorsolateral prefrontal cortex, pre‐supplementary motor area (pre‐SMA), cerebellum, and primary somatosensory cortex (S1) [4, 5, 6]. Critically, STDT dynamically modulates during movement and is impaired in neurological disorders such as Parkinson's disease (PD), where its deterioration correlates with motor deficits [7, 8]. Emerging evidence suggests that motor training can enhance STDT through plasticity in S1 [9, 10]; however, the underlying regulatory mechanisms remain poorly understood. While several networks associated with motor planning, execution, prediction, and multisensory integration have been proposed to explain this enhancement, a systematic investigation into STDT's role in motor training is crucial to unraveling its contribution to sensorimotor plasticity.

Existing research on STDT and movement has largely focused on sensorimotor integration mechanisms in injury recovery and rehabilitation for neurological diseases. Sensorimotor integration refers to the central nervous system's process of incorporating sensory input to refine motor execution [11]. A landmark study demonstrated that STDT progressively worsens in PD, correlating with dyskinesia severity [12]. PD patients exhibit increased STDT during movement execution and within 100 ms post‐movement, whereas healthy individuals show STDT changes even at 200 ms post‐execution, indicating a broader temporal modulation window [11, 13]. Studies on motor training and STDT regulation suggest that specific training techniques can modulate STDT. For example, short‐term “coin rotation” training significantly reduced STDT and improved motor scores in healthy participants [9]. Similarly, a four‐week training study found that both home‐based and supervised exercise programs enhanced STDT [14]. However, the extent of improvement appears to vary based on training modality. For instance, power grip movements (grasping with all fingers flexed) induce greater STDT changes than index finger abduction or two‐finger grasping (thumb‐index pinch) [15]. While these findings suggest that motor training influences STDT via sensorimotor integration mechanisms, the underlying neural mechanisms remain unknown.

However, actual motor execution is not always feasible, nor is it the optimal approach for training. For example, in stroke patients with movement disorders, motor imagery (MI) has been shown to produce equal or even superior rehabilitation outcomes compared to physical execution [16, 17]. MI, defined as'the mental representation of oneself performing a motor act without overt movement [18]' has gained increasing attention in rehabilitation because it does not require physical execution. Numerous studies have demonstrated that MI not only enhances kinematic performance in patients but also provides effective training benefits for healthy individuals [19, 20]. Additionally, kinematic analyses have shown that combining actual execution training with MI can significantly improve motor planning abilities in children with mild cerebral palsy. This improvement is reflected in faster and more direct target‐oriented movements [21]. Recent advancements in Human Activity Recognition (HAR) [22], especially label‐free pose estimation tools like DeepLabCut [23, 24], have enabled high‐accuracy video analysis of complex motions, offering valuable applications for hand kinematic research [25, 26]. Studies on kinematic performance without physical representation in actual execution training suggest that when participants imagine difficult movements, their MI speed (evaluated through mental timing) is slower than actual execution [27]. However, the specific kinematic differences between actual execution and MI remain unclear. Beyond movement, somatosensory perception, which is essential for processing tactile and proprioceptive inputs, significantly contributes to MI [28]. Recent studies indicate that MI can activate the somatosensory system [29] and modulate the excitability of the primary somatosensory cortex (S1) [30, 31]. While MI does not appear to increase STDT [13], similar to actual execution, research suggests that verbal cues can reduce STDT, indicating that cognitive strategies may bypass overt movement to influence STDT processing [32]. Given the crucial role of STDT in actual execution training and motor performance, it is essential to investigate the extent to which MI modulates STDT.

Electroencephalography (EEG), owing to its extremely high temporal precision, can detect early activity signals in motor and sensory research, such as somatosensory‐evoked potentials (SEP). SEP, extracted in various scenarios, allows researchers to explore immediate alterations in S1 following sensory activities after motor training [33]. Several studies have demonstrated that repetitive synchronized movements lead to plastic changes not only in the primary motor cortex (M1) [34] but also in S1 [35]. Specifically, the N20‐P25 amplitudes of the SEP signal, which represent components generated in S1, are enhanced after learning a repetitive motor task [36]. A similar result has also been observed in MI. Participants who engaged in visually guided MI exhibited a greater increase in the N20‐P25 component [37]. On the other hand, several studies have also found a strong correlation between SEP and STDT. Both the rise in STDT due to dystonia [8] and S1‐targeted interventions [5] have shown a significant correlation with an increase in N20‐P25. In addition to temporal somatosensory changes occurring during motor training or movement disorders, the spatial somatosensory domain (specifically, the spatial inhibition ratio, SIR) also changes at specific times [8]. For instance, movement deficits have been linked to impaired orientation sensitivity at the fingertips [38]. Therefore, SEP effectively reveals the neural mechanisms underlying sensorimotor integration and STDT changes following motor training. Although kinematic analysis has long been used in rehabilitation and motor training, only recent studies have begun to explore the potential relationship between STDT ability and kinematic parameters. While STDT changes in PD patients are not directly related to movement amplitude or speed, they are associated with increased variability in finger‐tapping amplitude and speed [39].

Therefore, to investigate the effects of different hand‐training techniques on STDT and kinematic performance, we designed four training conditions: ball‐rotation training (BRT) for actual execution, tactile control (Tactile) for simple gripping, visual‐guided imagery (VGI) for MI without movement execution, and a control group (Control) for baseline conditions without intervention. To ensure STDT measurement accuracy, we developed a new assessment platform, refined the protocol using the staircase method, and conducted STDT assessments before and after training for each participant. Additionally, we recorded resting‐state electroencephalograms (rsEEG), SEP, and video data during motor testing. These recordings were used for subsequent power spectrum analysis, evaluation of temporal and spatial somatosensory plasticity, and kinematic assessments. In this study, we aimed to (1) explore differences in kinematic performance between actual execution training and MI; (2) investigate whether motor training, particularly MI, can improve STDT ability; and (3) identify the SEP‐related brain mechanisms underlying variations in kinematic performance and STDT across motor training techniques, as well as determine how plasticity is induced in somatosensory‐related brain regions.

## 2 Materials and Methods

### 2.1 Participants

For this study, the study sample size was estimated using G*power (version 3.1.9.7) [40]. To achieve statistical significance, with an effect size of 0.25, a power of 0.8, and an *α*‐error of 0.05, the minimum number of total participants was established in *n* = 12 per group (48 total subjects). Therefore, 48 neurologically healthy participants (25 males, 23 females, aged 23.64 ± 1.84 years), all right‐handed measured based on the Edinburgh Handedness Inventory (EIH) [41], were recruited from the Beijing Institute of Technology. Participants were randomly divided into four groups (*n* = 12 per group). All the participants had normal intelligence and none of the following conditions/experiences: (a) history of mental illness, (b) brain damage caused by the long‐term use of drugs, or (c) taking drugs with actions on the CNS level at the times of the experiments or before the experiment at least 1 month. The experiment was conducted with the understanding and written informed consent of each participant, conforming to the Declaration of Helsinki and the standards established by the Institutional Review Board and Ethics Committee of the Beijing Institute of Technology. The experimental protocol was approved by the Institutional Review Board and Ethics Committee of the Beijing Institute of Technology and carried out in accordance with relevant guidelines and regulations. The basic dimensions of the hand were measured by each participant before the experiment, including hand length and palm width.

### 2.2 Experimental Procedure

**FIGURE 1:**
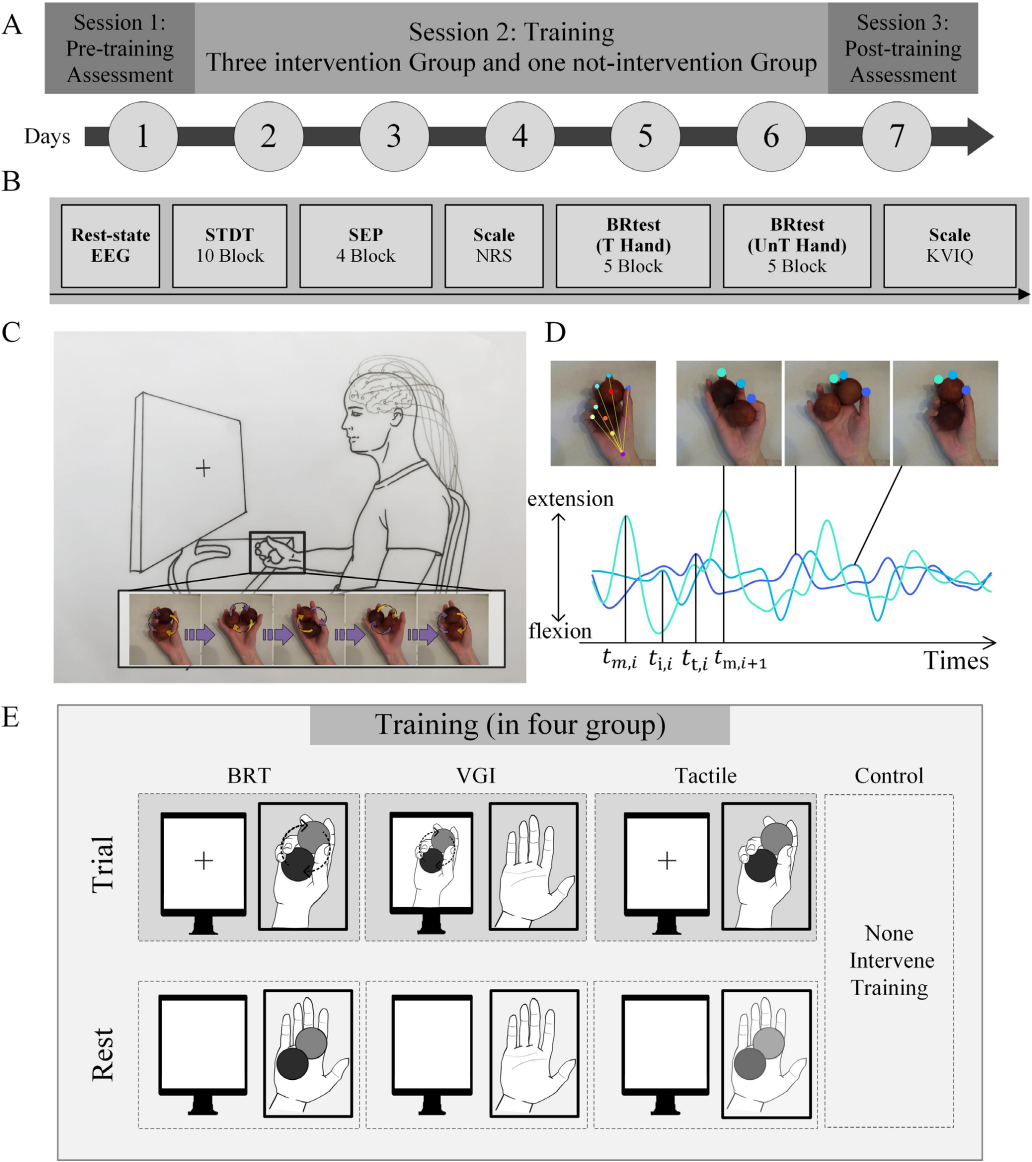
Experimental flow chart and motor paradigm. (A) Overall experimental procedures, including pre‐training assessment (baseline, 1 day), motor training (5 days), post‐training assessment (1 day); (B) Procedure for assessment days, including 5‐min rsEEG (for power spectrum analysis), STDT, SEP, and NRS for Intensity of electrical stimulation, BR test (right and left, in order), KVIQ; (C) motor paradigm; (D) Kinematics analysis. For the sake of simplicity, the diagram shows only the trajectories and phase differences between thumb and index finger of one participant; (E) Training paradigm of each group, including BRT for actual execution, Tactile for simple gripping, VGI for MI without movement execution, and Control for baseline conditions without intervention.

Each participant underwent a seven‐day protocol consisting of two assessment days (pre‐training assessment on day one and post‐training assessment on day seven) and five training days (days two to six), as shown in Figure 1A. The assessments and training were conducted in separate rooms under the supervision of different experimenters.

On assessment days, participants completed static EEG recording, STDT testing, SEP recording, ball rotation testing, and two scales: the electrical stimulation intensity scale of Numeric Rating Scale (NRS) for Intensity of electrical stimulation and the Kinaesthetic Vividness Inventory Questionnaire (KVIQ). The experimental procedure is illustrated in Figure 1B.

The ball rotation movement was chosen for both training and assessment, as shown in Figure 1C. A pair of identical wooden balls (40 mm diameter, 50 ± 2 g) was used. The training focused on the participants' right hand (trained hand) while both hands underwent testing. Participants were instructed to rotate the balls clockwise with their right hand and counterclockwise with their left hand, ensuring as stable a rotation as possible (i.e., minimizing interruptions or dropping the balls).

Participants were randomly assigned to one of four groups, as shown in Figure 1E: BRT (actual execution training), Tactile (simple gripping training), VGI (visual‐guided imagery), and Control (baseline condition without intervention). In the selection of the motor imagery paradigm, to avoid the influence of kinesthetic perception, we did not choose the recently proposed dynamic motor imagery (dMI), but instead selected the static motor imagery (sMI), which is adopting a congruent body position and embodying spatial and temporal features of the movement without entirely performing it [42]. On training days, participants, except for those in the control group, participated in their designated training, completing 10 blocks of 2 min each, with a 1‐min break between blocks. In all three training groups, participants were trained while keeping their right hand inside an obscured box and focusing on screens. In BRT, participants performed ball rotation, maintaining stability and avoiding dropping the ball. In Tactile, participants held the ball gently without rotating it. In VGI, participants kept their right hand steady and watched a video of their own ball rotation movement, edited from pre‐training day recordings. For the control group, 12 participants were informed after the first day of the trial that they would not undergo training for the next 5 days. Participants in both the VGI and control groups were instructed to avoid any ball rotation‐like activities until the next assessment day.

### 2.3 Behavioral and Motor Task

The behavioral tests included two tasks: the ball rotation test (BRtest) and the STDT test.

#### 2.3.1 BR Score

A BRtest was conducted to measure the effects of training across different groups. Participants performed the BRtests with both right and left hands, and each tested five times for 20 s per trial. During each trial, participants were instructed to rotate the balls as quickly as possible. Each time the balls swapped positions, it was counted as one rotation, and the total number of rotations (T) was recorded. If a ball fell on the table, the participant had to pick it up quickly and continue the test, with the number of drops recorded as D. If a ball fell on the ground, the participant had to stop the test. After five trials, an additional test was conducted, with the number of retests recorded as R.

The BR scores for each hand were calculated separately as follows:

(1) $$\mathrm{BR}\;\text{Score}=\frac{\left(1-0.1\times \frac{D}{N_{\text{Block}}}\right)\times \sum_{i=1}^{\text{Block}}T_{i}}{N_{\text{Block}}+0.5\times R}$$

#### 2.3.2 Kinematic Analysis

A high‐speed camera (frame rate 100 Hz, video resolution, 1280 × 720) recorded hand movements during the BR test. The camera was positioned 0.5 m above the participant's hand. Data analysis utilized an open‐source, markerless pose estimation toolbox and deep neural networks (DeepLabCut: https://www.mackenziemathislab.org/deeplabcut) [23, 24]. Nine markers were manually labeled in the training set at the tips of five fingers, the centers of the two balls, the palm, and the wrist, and five skeletons automatically connected between the wrist and five finger markers, as shown in Figure 1D.

We used the default algorithm (k‐means) to extract and label 3860 frames (including 20 screenshots for each participant, each test day, and each hand) as the initial training set, and gradually supplemented it to 10,000 frames from the video set with poor performance for merging datasets. Subsequently, the ResNet‐50 is used for model iteration. Other parameters are as follows: Training Fraction = 0.8, batch size = 8, using the imgaug augmentation strategy, max iters = 1,030,000, pcutoff = 0.6.

Then, we evaluated the model and ensured that the results met the requirements (the mean pixel error of the training set = 8.39 ± 0.08, and that of the test set = 9.79 ± 0.07). The resulting time‐series data were processed using a 0.5–5 Hz second‐order Butterworth bandpass filter, and individual undesirable data with a likelihood less than 0.75 were excluded.

To evaluate finger movement speed, we calculated the time difference between the maximum extension of each finger during two consecutive cycles (i.e., one cycle) and then computed the average cycle per block. For cross‐day comparisons, we calculated the average number of finger movement cycles on each measurement day.

To assess differences in finger coordination patterns, we calculated the phase differences for each finger combination (thumb‐index, thumb‐middle, index‐middle, etc.) during movement, using these as the indicators of coordination patterns. First, the relative phase (φ) for each participant was calculated for each block for all finger combinations (f and h):

(2) $$\varphi_{\mathrm{hf}}=\frac{t_{f,i}-t_{h,i}}{t_{m,i+1}-t_{f,i}}$$

where, $t_{f,i}$ and $t_{h,i}$ represent the times of the corresponding fingers at the *i*th peak. The relative phase φ ranges from 0 to 1, where 0 indicates the fingers are perfectly in phase, and 0.5 means antiphase. To unify the representation of phase alignment, we carried out a normalized remapping of phase differences. This processing converts the original phase difference range [0, 1] (where 0.5 denotes anti‐phase) to a normalized range [0, 1] (where 1 denotes anti‐phase), while discarding the temporal order of phase progression (e.g., leading or lagging). The median relative phase was calculated for each block. Finally, we calculated the phase variation (i.e., the difference between the maximum and minimum relative phase values among the five blocks) for each finger combination on each test day, as well as the overall phase variation across all finger combinations.

#### 2.3.3 STDT

**FIGURE 2:**
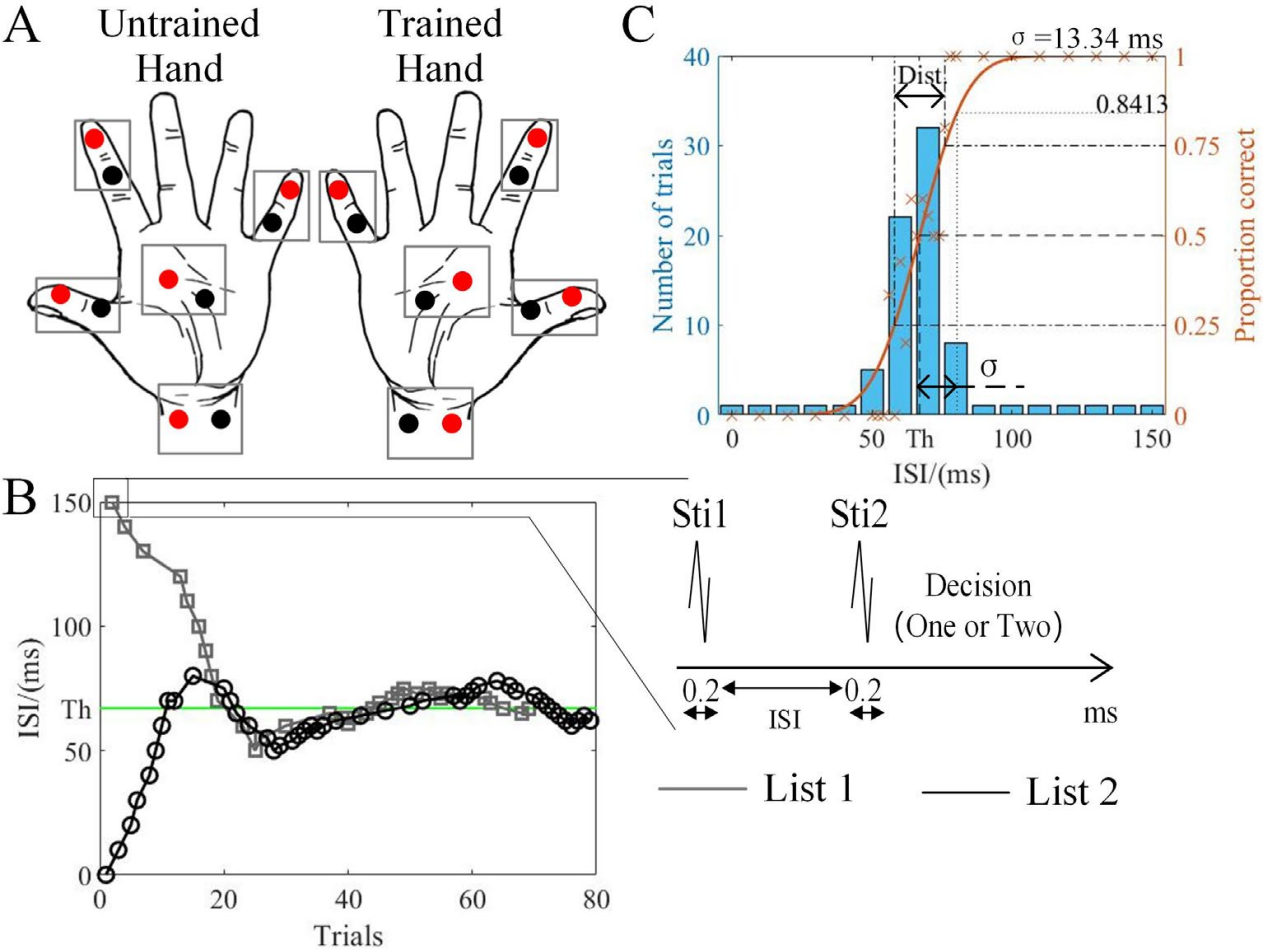
Improved STDT program diagram. (A) assessment locations, including index finger, thumb finger, little finger, palm, and wrist in each hand; (B) Example of a random staircase by one participant; (C) The corresponding psychometric curve. The bar chart shows the number of repetitions for each ISI (left scale). The curve shows the fitted function through the measured data points (right scale). Dist. is short for distance, which is the distance between 25% and 75% threshold.

The STDT was measured using the Double‐Random Staircase method, as shown in Figure 2. Ten assessments were performed, specifically measuring the thumbs, index fingers, small fingers, palms, and wrists of each hand, as shown in Figure 2A. In each assessment, the stimuli followed two sequences, starting at a maximum inter‐stimulus interval (ISI) of approximately 150 ms and a minimum ISI of about 0 ms. Each sequence computed the ISI separately and presented them randomly after mixing, as shown in Figure 2B. During the assessment, participants were required to respond to each perceived stimulus by choosing between “one stimulus' and” two stimuli. If a participant reported feeling ‘one stimulus' during a trial, the next stimulus in that sequence increased by one step. Conversely, if the participant reported “two stimuli,” the next stimulus in that sequence decreased by one step. If the participant's response to the current trial was inconsistent with the previous response (e.g., if they answered ‘one stimulus' in the last trial but “two stimuli” in the current trial), the current trial was recorded as a “Reversal.” When a participant reached two reversals, the step size was halved. The initial step was 10 ms, which was then successively reduced to 5 ms, 2 ms. Once the step size reached 2 ms, the reversal count began. A sequence was considered complete when the participant reached eight reversals. The assessment ended when both sequences reached eight reversals or when the total number of trials reached 110. To ensure response accuracy, up to ten catch trials with a single stimulus were randomly included in each assessment.

For each assessment, using MATLAB (MathWorks Version 2022b, Natick, MA, USA), the proportion of “two stimuli” responses was calculated for each ISI. Then, all results were fitted to a cumulative Gaussian distribution using the maximum likelihood method [43, 44], as shown in Figure 2C.

(3) $$f\left(x\right)=\frac{1}{2}\left[1+\mathrm{erf}\left(\frac{x-\mathrm{Th}}{\sigma \sqrt{2}}\right)\right]$$

Here, *σ* describes the steepness of the curve, and Th corresponds to the 50% threshold (i.e., discrimination threshold, referred to as “threshold”) [45]. The coefficient of determination (*R* ^2^) was measured using a least squares fitting program to verify whether the psychometric function conformed to a cumulative Gaussian distribution. Finally, the 25% and 75% thresholds were determined from the fitting curve, and the distance between them was calculated as the discrimination threshold (referred to as “distance”). These two measures—threshold and distance—characterize the assessment results.

### 2.4 EEG Recording and Processing

#### 2.4.1 EEG Recording

Our EEG recording and preprocessing procedure strictly follows the guidelines by Pernet et al. [46]. For each participant, rsEEG and SEP were collected, respectively, for subsequent analysis. On the assessment day, participants were seated in a room sheltered from light and electromagnetic interference and instructed to close their eyes and relax to record EEG data. To obtain the rsEEG, data was recorded using a 64‐electrode Neuroscan SynAmps2 amplifier, following the International 10–20 System, before participants took part in other assessments for 5 min. The reference electrode was placed between Oz and CPz, and the ground electrode was placed on AFz. EEG signals were sampled at 1000 Hz, and the impedances of the electrodes were maintained below 10 kΩ.

#### 2.4.2 EEG Preprocessing

Preprocessing and power spectral analysis of EEG data were conducted using the open‐source MATLAB toolbox EEGLab v2022.0 [47]. The obtained data were first re‐referenced offline to the average signal across all electrodes, bandpass filtered between 1 and 80 Hz, and additionally filtered with a 50 Hz notch filter to reduce line noise artifacts. After filtering, the data were segmented into 2‐s epochs, and any epochs with an amplitude exceeding ±80 μV at any electrode were rejected. Finally, an independent component analysis (ICA) was conducted, and components involving eye blinks or other muscle contraction artifacts, which were distinguished from brain‐originated signals, were subtracted based on amplitude and frequency characteristics.

#### 2.4.3 Power Spectrum

The band power was then computed to compare the power spectra in all trials. A discrete fast Fourier transform using the Welch method was employed as the calculation method for each epoch. The frequency range was divided into delta (1–4 Hz), theta (4–8 Hz), alpha (8–13 Hz), beta (13–30 Hz), and gamma (> 30–80 Hz) bands. Finally, the electrodes were classified and averaged according to brain regions: prefrontal (Fp1, Fpz, Fp2), left frontal (AF3, F7, F5, F3, F1), right frontal (AF4, F8, F6, F4, F2), left central (FC5, FC3, FC1, C5, C3, C1), right central (FC6, FC4, FC2, C6, C4, C2), left parietal (CP3, CP1, P3, P1), right parietal (CP4, CP2, P4, P2), left temporal (FT7, T7, TP7, CP5, P7, P5), right temporal (FT8, T8, TP8, CP8, P8, P8), and occipital (PO7, PO5, PO3, POz, PO4, PO6, PO8, O1, Oz, O2).

#### 2.4.4 SEP

SEPs were recorded using Ag–AgCl surface electrodes with a Neuroscan SynAmps2 amplifier. The active electrode was placed at CP3 and the reference electrode at Fz, following the international 10–20 system [48], and the impedances of the electrodes were maintained below 10 kΩ. The digital nerves of the right thumb (T) and index finger (I) were stimulated with a Digitimer DS7A constant‐current stimulator. The cathode was placed at the base of the first phalanx, and the anode 2 cm distally [49]. Monophasic square‐wave pulses (200 ms duration) were delivered at 250% of the sensory threshold with a stimulation frequency of 5 Hz. Recordings were collected at a sampling rate of 5 kHz, beginning 20 ms before stimulus onset and lasting 100 ms. Data were bandpass‐filtered from 3 kHz to 2 kHz. In the first block, 1000 sweeps were averaged, and the N20 peak latency and N20–P25 peak‐to‐peak amplitude were measured. A recording block of 750 frames was used to measure the N20–P25 recovery cycle. In this block, 750 trials were averaged, and paired pulses at ISI of 5, 20, and 40 ms were delivered. In the paired‐stimulus trials, responses after the second stimulus were obtained by subtracting the SEP waveform elicited by the first stimulus. The ratios R5, R20, and R40 were then defined as the response ratios between the second and first stimuli. Finally, two additional blocks of 750 trials were recorded, the first stimulating the right thumb only and the second stimulating both the thumb and index finger concomitantly by giving two simultaneous stimuli delivered through two constant‐current stimulators [50, 51]. The SIR of N20–25 was calculated as the ratio TI/(T + I) × 100%, where TI is the SEP amplitude obtained by simultaneous stimulation of the thumb and index finger, named as SIRdp, and T + I is the arithmetic sum of the SEP obtained by the individual stimulation of the two fingers, named as SIRsum.

### 2.5 Statistical Analysis

For all results, the Shapiro–Wilk test and Bartlett test were performed using the R package rstatix to verify the normality and homogeneity of variances. Since the data did not fully meet normality and homogeneity assumptions, nonparametric methods were used for statistical analysis.

For demographics, the Kruskal–Wallis test was conducted using the rcompanion package in R. If a significant effect was found, post hoc analysis was performed using the Bonferroni‐corrected paired Wilcoxon rank‐sum test, and the effect size is measured by the rank‐binary correlation coefficient (*r*).

For motor cycles from the kinematic analysis, motor scores, STDT at each location, SEP, SIR, and power spectrum on each assessment day, the Scheirer‐Ray‐Hare test (group × day) was conducted using the rcompanion package in R. If a significant effect was found, post hoc analysis was performed using the Bonferroni‐corrected paired Wilcoxon rank‐sum test, and the effect size was measured by the rank‐binary correlation coefficient (*r*).

Differences in digit coordination patterns across assessment days were analyzed using the Scheirer‐Ray‐Hare test (group × day). Additionally, total coordination patterns within each group were evaluated using the Scheirer‐Ray‐Hare test (finger combination × day) to compare each finger with the others. A post hoc analysis using the Bonferroni‐corrected paired Wilcoxon rank‐sum test was conducted when necessary.

To explore the relationship between behavioral results (motor score, STDT, and digit coordination pattern) and neural mechanisms (SEP and power spectrum), a linear model analysis was fitted for each group. To ensure consistency in calculations, the *x*‐axis (behavioral results) and *y*‐axis (SEP or power spectrum results) were represented as the rate of change.

## 3 Result

### 3.1 Statistical Comparison of Demographics and Scale

With regard to demographics, we did not find significant differences in gender, age, basic dimensions of hand (hand length and palm width), and the scores (KVIQ and the EIH) across four groups, as shown in Table S1.

### 3.2 Statistical Comparison of Overall Kinematic Performance and STDT

**FIGURE 3:**
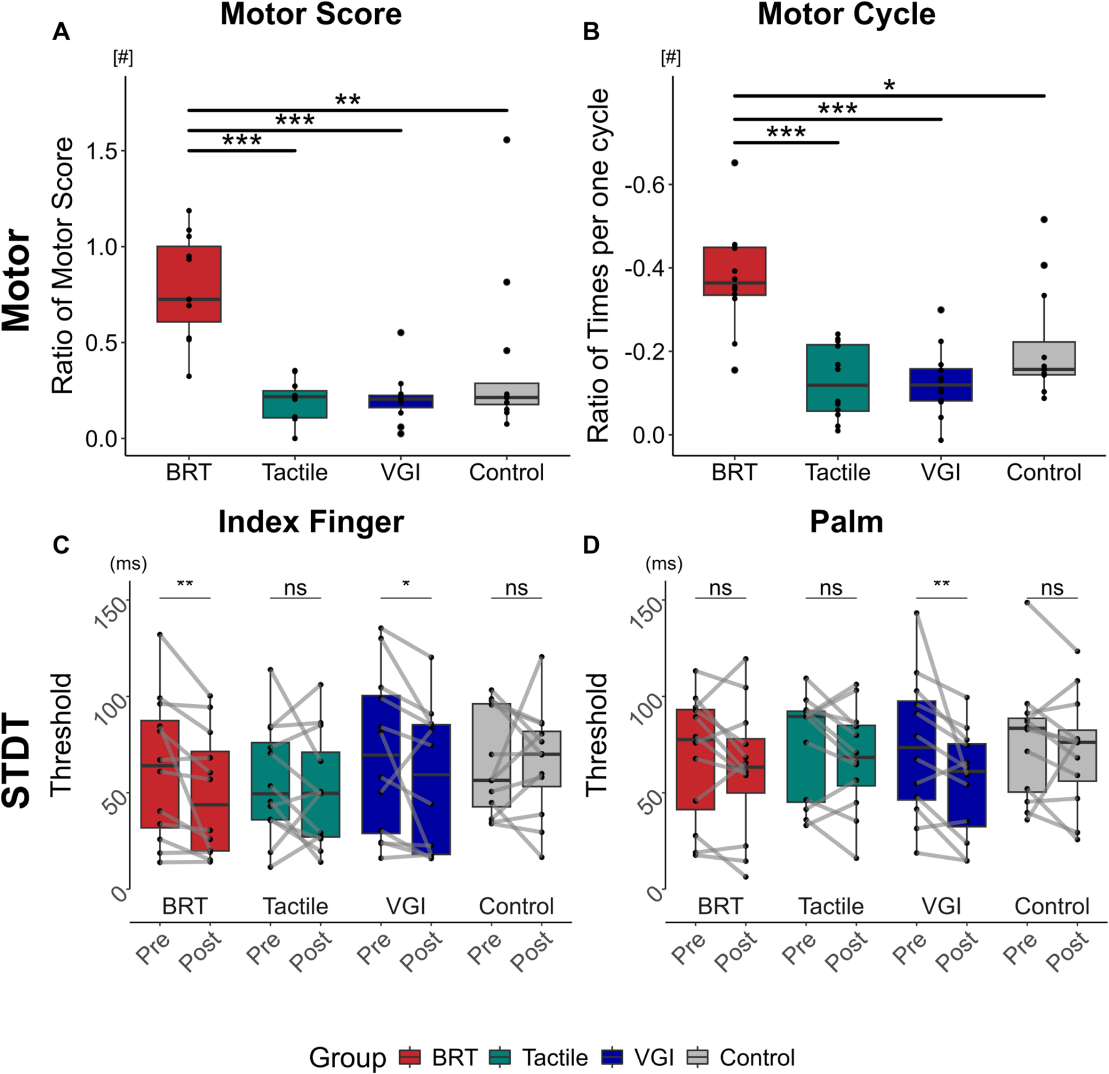
Effects of training technique (BRT, Tactile, VGI, Control) on motor performance and the threshold of STDT on trained hand. (A, B) The box plot shows the ratios of motor scores and motor cycle (normalized to baseline) in the BRT, tactile, VGI, and Control on trained hand. (C, D) The box plot compared pre‐ and post‐training of the threshold of STDT at the index finger and palm in each group on trained hand. The connecting lines show individual group trends. Data are shown as the median [95% confidence interval, CI] (*p < 0.05, **p < 0.01, ***p < 0.001, ^ns^ *p* > 0.05). Specific values are shown in Tables S2 and S3.

The data were rejected by the Shapiro–Wilk test for the normal hypothesis for motor score (*W* = 0.83, *p* = 0.022 in BRT, trained hand, and *W* = 0.78, *p* = 0.005 in Control, trained hand), motor cycle (*W* = 0.68, *p* = 0.0006 in BRT, untrained hand, *W* = 0.83, *p* = 0.022 in Tactile, trained hand, and etc.), and STDT (*W* = 0.77, *p* = 0.004 in BRT, right index finger, *W* = 0.76, *p* = 0.003 in Tactile, left little finger, *W* = 0.82, *p* = 0.018 in VGI, right thumb finger, *W* = 0.83, *p* = 0.021 in Control, right little finger, and etc.), so a non‐parametric method was adopted. Scheirer‐Ray‐Hare analysis revealed significant differences in both motor performance and STDT across intervention groups and assessment days. For the trained side, motor score and motor cycle from the kinematic analysis showed significant main effects of group (motor score: *χ* ^2^ = 17.9600, *p* < 0.001 and motor cycle: *χ* ^2^ = 18.0418, *p* < 0.001) and day (pre vs. post; motor score: *χ* ^2^ = 13.8586, *p* < 0.001, motor cycle: *χ* ^2^ = 15.1980, *p* < 0.001), but no significant group × day interaction (motor score: *χ* ^2^ = 7.0762, *p* = 0.069, motor cycle: *χ* ^2^ = 6.3095, *p* = 0.097). For the untrained side, motor score and motor cycle showed a significant main effect only between groups (motor score: *χ* ^2^ = 10.7303, *p* = 0.001 and motor cycle: *χ* ^2^ = 11.4107, *p* < 0.001), but no significant effect of day (motor score: *χ* ^2^ = 4.6351, *p* = 0.200, motor cycle: *χ* ^2^ = 4.0719, *p* = 0.254) and interaction(motor score: *χ* ^2^ = 1.1394, *p* = 0.768, motor cycle: *χ* ^2^ = 0.7872, *p* = 0.852). Post hoc comparisons revealed distinct variations in motor performance within and across the intervention groups. Participants in all four groups showed significant within‐group differences and large effects across training sessions, as shown in Table S1. However, only the BRT group demonstrated a significant difference compared to the other three groups on the trained side. As illustrated in Figure 3A, the improvement rate of the motor score in the BRT group was significantly higher than in the other three groups (BRT vs. tactile, 0.83 [0.61, 1.07] vs. 0.21 [0.10, 0.25], *p* < 0.001, effect size of *r* = 0.82; vs. VGI, 0.21 [0.10, 0.22], *p* < 0.001, effect size of *r* = 0.81; vs. Control, 0.21 [0.17, 0.34], *p* = 0.004, effect size of *r* = 0.64). Similarly, the reduction rate of times per cycle in the BRT group, as obtained through kinematic analysis, was significantly greater than that in the other three groups (BRT vs. tactile, −0.37 [−0.45, −0.34] vs. −0.16 [−0.21, −0.06], *p* < 0.001, effect size of *r* = 0.75; vs. VGI, −0.12 [−0.16, −0.08], *p* < 0.001, effect size of *r* = 0.75; vs. Control, −0.16 [−0.26, −0.14], *p* = 0.022, effect size of *r* = 0.54), as shown in Figure 3 (B). On the untrained side, only the motor score improvement rate was significantly higher in the BRT group than in the tactile group (0.41 [0.21, 0.74] vs. 0.13 [0.05, 0.29], *p* = 0.033, effect size of *r* = 0.55), as shown in Table S2 and Figure S1A,B.

For somatosensory function, STDT showed distinct patterns in the Scheirer‐Ray‐Hare analysis: On the trained side, significant day‐dependent reductions were observed in the 50% threshold of the index finger (*χ* ^2^ = 4.4536, *p* = 0.035) and palm (*χ* ^2^ = 3.9969, *p* = 0.045), but no significant group‐level differences were found (index finger: *p* = 0.948, palm: *p* = 0.11). For both palms, the distance between 25% and 75% thresholds exhibited significant day effects (trained side: *χ* ^2^ = 13.8020, *p* < 0.001; untrained side: *χ* ^2^ = 7.1143, *p* = 0.007), but no significant group‐level differences were found (*p* > 0.05). Post hoc comparisons showed distinct changes in the BRT and VGI. A marked reduction in STDT for the index finger was observed in the BRT (pre vs. post, 64.02 [29.83, 90.34] vs. 43.75 [19.62, 74.74], *p* = 0.007, effect size of *r* = 0.75) and VGI (pre vs. post, 69.57 [27.37, 101.84] vs. 59.38 [17.98, 85.47], *p* = 0.034, effect size of *r* = 0.61) groups on the trained hand. A similar trend was noted in the palm measurements, with the VGI group showing a significant improvement in the STDT (73.43 [44.70, 99.32] vs. 61.13 [29.59, 76.08], *p* = 0.003, effect size of *r* = 0.79), as shown in Figure 3C,D. No significant STDT changes were observed in the BRT and VGI groups for the index finger or palm on the untrained side, except for the palm distance on the trained and untrained hands of the VGI group, as shown in Table S3 and Figure S1C–F.

### 3.3 Digit Coordination Pattern of Each Group During Movement

**FIGURE 4:**
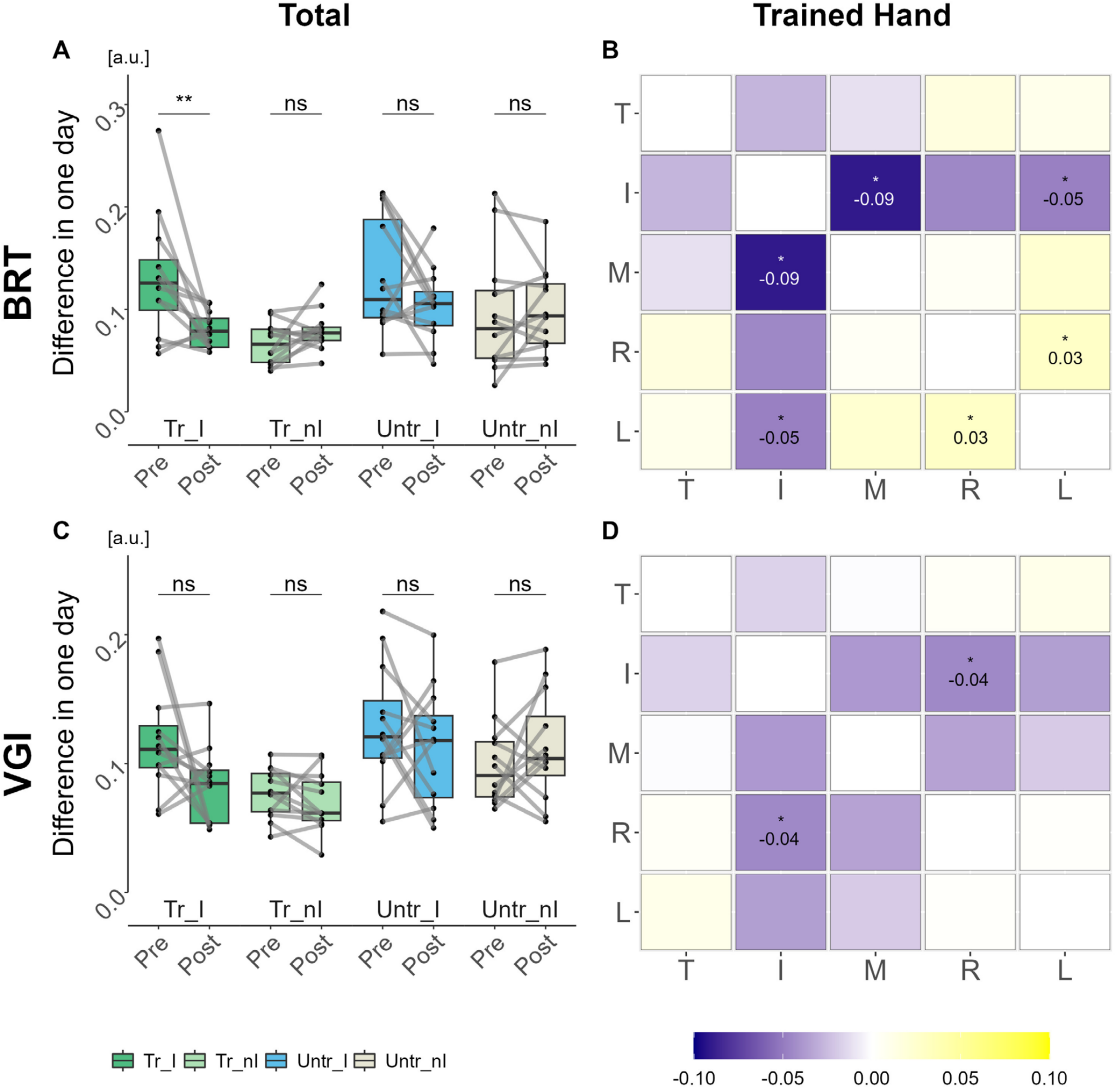
Difference in digit coordination patterns on total and trained hand. (A, C) The box plot compared pre‐ and post‐training of the overall digit coordination patterns across hand based on index finger in BRT and VGI. The connecting lines show individual group trends. (B, D) Difference in digit coordination patterns, combination of digits across assessment days. T = Thumb finger, I = Index finger, M = Middle finger, *R* = Ring finger, L = Little finger. Zero indicates a coordination pattern that is identical across two assessment days. Data are shown as the median [95% CI] (*p < 0.05, **p < 0.01, ***p < 0.001, ^ns^ *p* > 0.05). Specific values are shown in Tables S4 and S5.

The data were rejected by the Shapiro–Wilk test for the normal hypothesis for Digit Coordination Pattern in some finger paired (*W* = 0.76, *p* = 0.003 in VGI based on non‐index finger, trained hand, and *W* = 0.82, *p* = 0.015 in Control based on index finger, trained hand); so a non‐parametric method was adopted. The Scheirer‐Ray‐Hare analysis revealed significant main effects in the overall digit coordination patterns across hand (BRT: *χ* ^2^ = 17.7800, *p* < 0.001, Tactile: *χ* ^2^ = 19.4232, *p* < 0.001, VGI: *χ* ^2^ = 17.8057, *p* < 0.001, Control: *χ* ^2^ = 9.4582, *p* = 0.023) based on index finger but not based on others. However, a significant effect of day was observed only in the BRT group (*χ* ^2^ = 4.0902, *p* < 0.0431) in the overall digit coordination patterns based on index finger. Post hoc comparisons revealed significant variations in the index finger of the trained hand (pre vs. post, 0.13 [0.11, 0.06] vs. 0.08 [0.06, 0.09], *p* = 0.005, effect size of *r* = 0.75), as shown in Figure 4A,C, while additional results are presented in Table S4.

To determine whether the digit coordination pattern includes all five digits or specific digit combinations, differences in 10 digit‐pair combinations (e.g., thumb‐index finger, thumb‐middle finger, ring‐little fingers, etc.) were analyzed. The coordination patterns found in the BRT and VGI groups are shown in Figure 4B,D, while additional results are presented in Table S5 and Figure S2.

### 3.4 Difference of SEP in Each Group Across Motor Training

**FIGURE 5:**
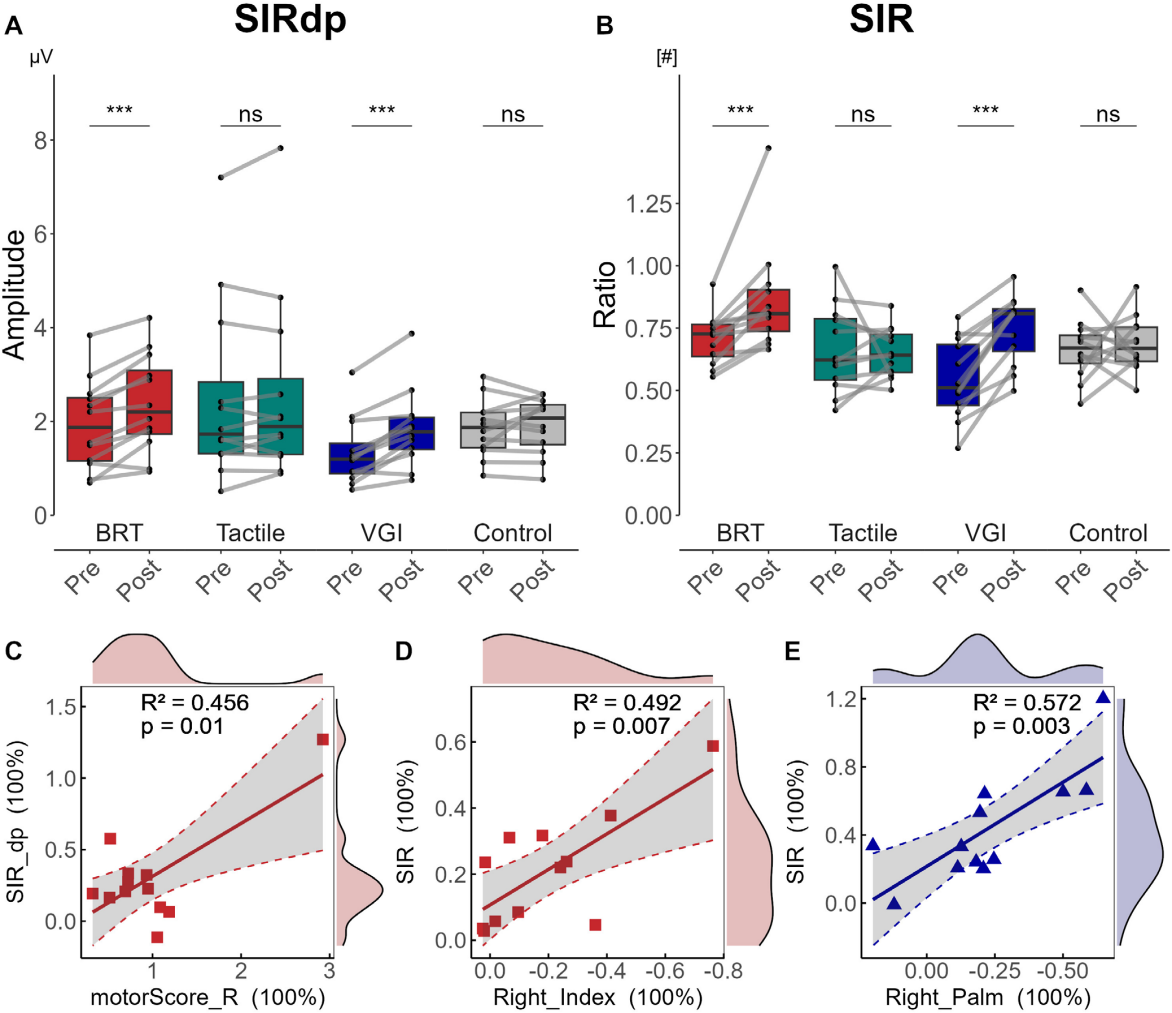
Changes in SEP in each technique and the relationship between SIR and behaviors. (A, B) The box plot compared pre‐ and post‐training of SIRdp (the SSEP amplitude obtained by simultaneous stimulation of the thumb and index finger) and SIR in each group. The connecting lines show individual group trends. (C–E) Relationship between motor score of the right hand and SIRdp in BRT, between STDT in the index finger of the right hand and SIR in BRT, and between STDT in the palm of the right hand and SIR in VGI. Data are shown as the median [95% CI] (*p < 0.05, **p < 0.01, ***p < 0.001, ^ns^ *p* > 0.05). Specific values are shown in Table S6.

The data were rejected by the Shapiro–Wilk test for the normal hypothesis for SEP (*W* = 0.75, *p* = 0.003 in BRT, *W* = 0.79, *p* = 0.002 in Tactile based on SIR, and etc.), so a non‐parametric method was adopted. Scheirer‐Ray‐Hare analysis revealed significant main effects in SIR for both groups (*χ* ^2^ = 9.7016, *p* = 0.021) and day (*χ* ^2^ = 7.0804, *p* < 0.007). However, for SIRdp, a significant effect was found only for group (*χ* ^2^ = 13.7132, *p* = 0.003). Post hoc comparisons showed distinct variations in BRT (pre vs. post, 1.87 [1.14, 2.53] vs. 2.2 [1.68, 3.20], effect size of *r* = 0.88 for SIRdp, 0.73 [0.63, 0.77] vs. 0.81 [0.73, 0.91], effect size of *r* = 0.86 for SIR, *p* < 0.001) and VGI (pre vs. post, *p* < 0.001, 1.2 [0.86, 1.69] vs. 1.78 [1.37, 2.10], effect size of *r* = 0.86 for SIRdp, 0.51 [0.43, 0.69] vs. 0.81 [0.64, 0.83], effect size of *r* = 0.86 for SIR) on the trained hand, as illustrated in Figure 5A,B, while additional results are presented in Table S6.

To investigate the relationship between STDT, motor performance, and SEP, we calculated the correlation coefficient. A positive correlation was observed between the motor score and SIRdp in the BRT group on the trained hand, between the index finger of STDT and SIR in the BRT group on the trained hand, as shown in Figure 5C–E.

### 3.5 Difference of Power Spectrum in Each Group Across Motor Training

**FIGURE 6:**
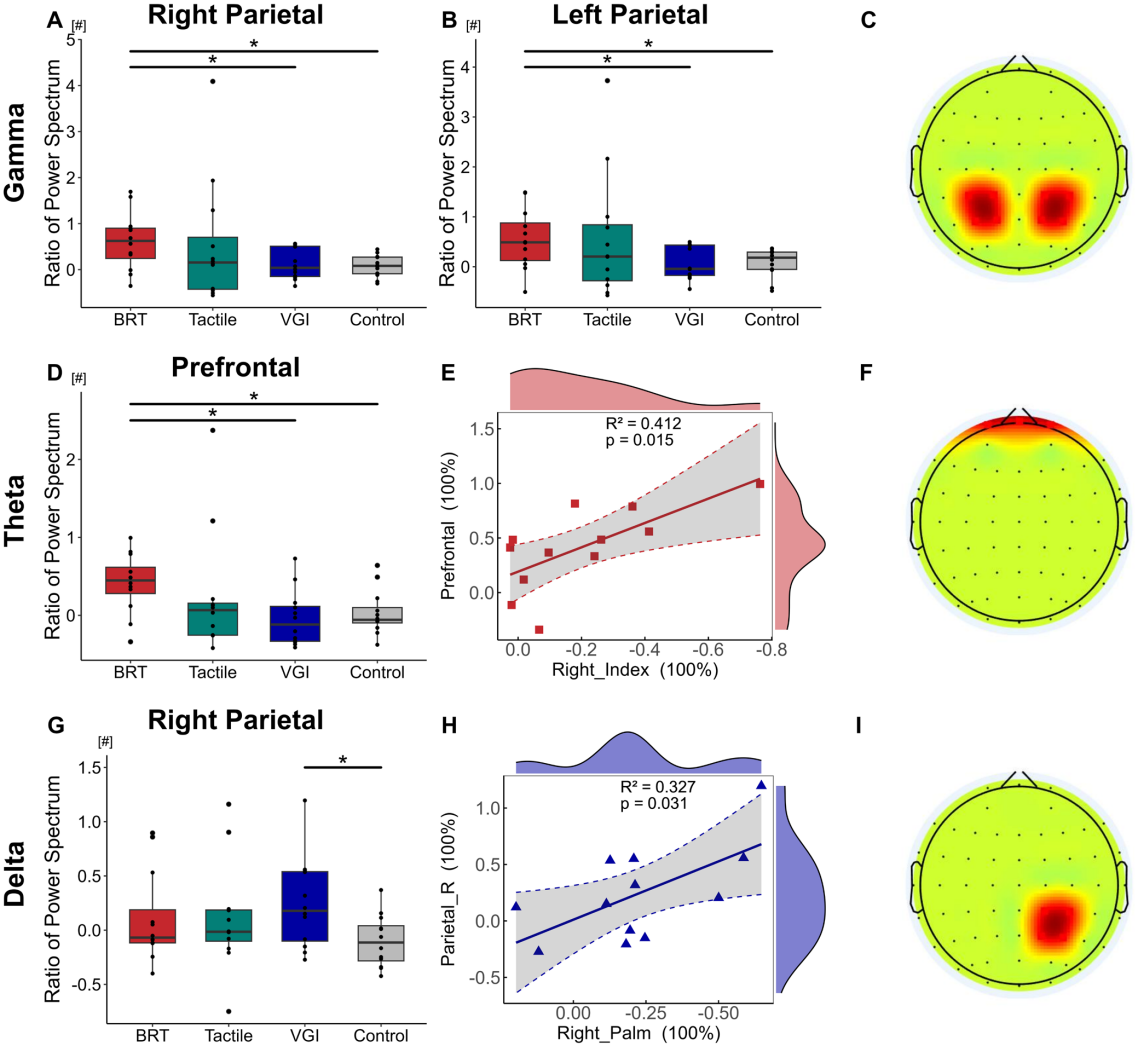
Changes in power spectrum in each technique and the relationship between power spectrum and behaviors. (A, B, D, G) The box plot shows the ratios of power spectrum (normalized to baseline) in the BRT, tactile, VGI, and Control in Right Parietal (including electrodes CP4, CP2, P4, P2), Left Parietal (including electrodes CP3, CP1, P3, P1), and Prefrontal (including electrodes Fp1, Fpz, Fp2) cortex. (E, H) Relationship between STDT in the index finger of the right hand and theta band power spectral density in the prefrontal cortex in BRT, and between STDT in the palm of the right hand and delta band power spectral density in the right parietal cortex in VGI. (C, F, I) Schematic diagram of electrode positions where differences exist. Data are shown as the median [95% CI] (*p < 0.05, **p < 0.01, ***p < 0.001, ^ns^ *p* > 0.05). Specific values are shown in Table S7.

The data were rejected by the Shapiro–Wilk test for the normal hypothesis for gamma in the left parietal region (*W* = 0.77, *p* = 0.004 in BRT, *W* = 0.73, *p* = 0.002 in Tactile, and etc.), so a non‐parametric method was adopted. Scheirer‐Ray‐Hare analysis revealed significant main effects in the power spectrum of the group in several frequency bands: gamma in the right parietal region (*χ* ^2^ = 11.3097, *p* = 0.010), gamma in the left parietal region (*χ* ^2^ = 14.7253, *p* = 0.002), theta of prefrontal (*χ* ^2^ = 9.6934, *p* = 0.0213), delta of right parietal (*χ* ^2^ = 10.0381, *p* = 0.018). Post hoc comparisons showed differences in the gamma band of the right parietal (BRT vs. VGI: 0.62 [0.16, 0.91] vs. 0.04 [−0.16, 0.52], *p* < 0.033, effect size of *r* = 0.44, vs. Control: 0.08 [−0.10, 0.27], *p* < 0.024, effect size of *r* = 0.46), the gamma of the left parietal (BRT vs. VGI: 0.49 [0.10, 0.94] vs. −0.04 [−0.18, 0.44], *p* < 0.028, effect size of *r* = 0.45, vs. Control: 0.18 [−0.06, 0.30], *p* < 0.033, effect size of *r* = 0.44), the theta of prefrontal (BRT vs. VGI: 0.45 [0.21, 0.80] vs. −0.12 [−0.34, 0.13], *p* < 0.012, effect size of *r* = 0.51, vs. Control: −0.06 [−0.12, 0.14], *p* < 0.024, effect size of *r* = 0.46), the delta of right parietal (VGI vs. Control: 0.18 [−0.12, 0.54] vs. −0.11 [−0.30, 0.07], *p* < 0.028, effect size of *r* = 0.45), as shown in Figure 6A,B,D,G. The regions with differences as shown in Figure 6C,F,I, while additional results are presented in Table S6.

To further explore the relationship between STDT, motor performance, and the power spectrum, we calculated the correlation coefficient. A positive correlation was found between the index finger STDT and the prefrontal cortex in the BRT group on the trained hand, as well as between the palm STDT and the prefrontal cortex in the VGI on the trained hand, as shown in Figure 6E,H.

## 4 Discussion

This study explored the effects of different training paradigms on STDT and their potential neural mechanisms. By designing an experiment that included actual execution, simple motor training, and MI as three training techniques, along with neurophysiological indicators such as resting‐state EEG and SEP, the following key findings were observed:

The training interventions elicited significant improvements in motor performance and neurophysiological correlates of tactile temporal discrimination. Each group demonstrated enhanced motor ability post‐intervention; yet only the actual execution group showed superior performance compared to other conditions and exhibited notable transfer effects to the untrained side. Kinematic analysis revealed pronounced differences in the BRT, particularly regarding interdigital phase synchronization during finger movements. Concurrently, both BRT and VGI exhibited significant reductions in STDT, indicating enhanced tactile processing temporal resolution, while no comparable improvements were observed in Tactile or Control. These behavioral enhancements were paralleled by neurophysiological changes, including significant improvements in SIR metrics and power spectrum characteristics that correlated with STDT improvements. Power spectral density analysis further identified distinct cortical regions and frequency bands associated with the observed tactile discrimination enhancements following BRT and VGI.

### 4.1 The Kinematics Performance of Overall and Each Digit Coordination Pattern

Many factors influence motor learning efficiency, including external factors (i.e., task‐related factors, such as the amount of stimuli to be processed and the way to make decisions) and internal factors (i.e., subject‐related factors, such as attention factors, time–space positioning, movement preparation, and movement monitoring) [52]. Perhaps due to a learning effect, all four groups showed improved kinematic performance after training. However, only the BRT group demonstrated a significant difference from the other three groups. This result indicates the irreplaceability of actual execution and that a more detailed and individualized training paradigm may be required to achieve a training effect closer to actual execution. Furthermore, the results of the kinematic analysis also showed that the index finger may be a key in BRT in the two‐ball rotation paradigm of the hand, but no finger showed similar importance in MI, which may further demonstrate the difference between actual execution and MI training.

A previous study analyzing motor performance and kinematics in real and reproductive movements during two‐ball rotation training found significant differences in speed and interfinger coordination patterns from baseline but only on day eight [27]. This suggests that in this motor paradigm, participants may reach a performance plateau after approximately 1 week of training. Another study found that actual execution modulated corticospinal excitability not only on the training side but also on the untrained side; while gripping exercise decreased excitability on the training side [53], which may explain why gripping training not only did not differ from the control group in motor performance but also did not show special coordination patterns in kinematic analysis. Both sensory input and movement learning are crucial components of motor training [54], reflected in the significant performance difference between BRT and the other three groups on the trained side. On the untrained side, although the BRT achieved the largest improvement, it still showed no significant difference from the other three groups, especially the control group. This suggests that motor transfer between limbs may not be related to motor learning and somatosensory information or that other important factors are needed.

MI has been proven to be an effective technique to promote motor training [55], but its enhancement effect varies across studies [56, 57]. Some studies have compared action observation and the difficulty level of video materials [20, 58, 59] and found that there is an optimal difficulty range for MI training. Additionally, action observation can also significantly improve the effect of MI training. Based on these studies, Guillot et al. summarized five imagery dimensions that should be considered [42]: (i) the vividness of MI (corresponding to the clarity and richness of the mental representation, such as visualizing each body part), (ii) the exactness of MI (reflecting the degree of similarity with the corresponding actual execution), (iii) the controllability of MI (i.e., the ability to manipulate, transform or maintain the imagery content over time), (iv) the temporal congruence between MI and actual execution, and (v) the ability to form appropriate and accurate mental representations of the movement. In our study, we asked each of the participants VGI questions after each day of training to ensure that they were performing MI training correctly. Unfortunately, although the VGI showed improved kinematic performance after training, it did not significantly differ from the control group on either the trained or untrained sides. Subsequently, they further discovered that motor imagery accompanied by movements (dMI) would bring about a greater enhancement of motor ability in many dimensions than sMI and thus result in motor performance almost identical to actual movement [60]. They further deduced that this difference in performance might differ from sMI in the manner these representational networks are being activated and refined [42]. A meta‐analysis of the effects of MI on tennis player performance found that while motor visualization can improve serve accuracy and technique, it did not affect either service speed or return accuracy [19]. One possible reason is that MI tends to produce subtle yet potentially significant improvements in performance, especially when applied to enhance or rectify specific technical elements of motor skills [61]. This may explain why the kinematic changes produced by MI are more uniform and also suggests that the kinematic performance of MI training is similar to the actual execution but not identical, especially in the interfinger movement associated with the index finger.

### 4.2 The STDT Improvement Across Training of Each Technique

Recent studies on PD have pointed out that sensory and motor training can indirectly influence motor and sensory performance, respectively [62, 63]. Based on this view, some researchers believe that the one‐step integration of sensory and motor information is the necessary basis for goal‐directed movements of the hand [54]. In addition, sensory input has also been shown to be important for recovery after a stroke [64]. Although it has been proven that neurological diseases with movement disorders, such as PD and myasthenia gravis, may be accompanied by abnormal STDT [4, 7, 65], few studies have been conducted on whether STDT changes during motor training in healthy people, especially when comparing different motor training techniques.

Conte et al. found that STDT was increased on the moving hand at movement onset and up to 200 ms thereafter, but not beyond this point [13]. Furthermore, Yoshida et al. found that motor training reduced STDT on the trained side but did not affect the untrained side [9], implying that the change in STDT comes from the regulation of the sensorimotor integration mechanism. However, the study by Korucu et al. challenges this conjecture [14]: patients with fibromyalgia syndrome who performed 4 weeks of aerobic exercise showed a decrease in STDT in their hands after training. Although similar results have not been fully replicated in the study by Yamazaki et al. [66], these findings suggest that the regulation of STDT by motor training is not entirely body‐part‐specific. Considering that STDT has been observed to vary across different body parts [67], it is meaningful to explore whether this difference is also reflected in motor training. Our study found that actual execution did not reduce the STDT equally across all hand positions on the trained side; rather, it only reduced STDT in the index finger. Although this result is consistent with our kinematic analysis, unfortunately, no significant correlation was found between them.

On the other hand, compared with the possible inter‐limb migration in motor performance, this phenomenon does not seem to exist in somatosensory perception. Some different types of studies have respectively explored the issue of the transfer of motor and somatosensory functions between limbs during training. A study on mirror therapy found that compared with actual execution, there were significant changes in the activation of contralateral somatosensory areas in motor imagery [68]. Another study discovered that such changes in somatosensory areas only existed during active learning but did not occur during passive movement [69]. These studies seem to suggest that the plastic changes in somatosensory perception may be manifested as inter‐limb migration before movement. However, a study on the inter‐limb induction of somatosensory electrical stimulation during motor training found that artificially added somatosensory stimulation did not further increase skill acquisition [70]. These studies further illustrate that there may be a more complex relationship between motor and perception, rather than merely a simple sequential or simultaneous occurrence.

A key question about MI is to what extent sensory information is involved in the training process [42, 71]. Although many studies have confirmed similar activation of the sensorimotor cortex during actual execution and MI [72, 73], the extent to which sensory input contributes and whether there are lasting plastic effects remains a question. For example, although both the motor simulation theory (MST) and the effects imagery model (EIM) assert that MI is multimodal, involving visual, auditory, and somatosensory experiences, their interpretations of its regulation differ. MST considers MI functionally equivalent to actual execution, but EIM focuses on higher cognitive associations [71]. In our study, we found that MI can reduce STDT. Given that evidence suggests that STDT perception within this temporal band is not influenced by memory formation and does not require cognitive control [74, 75], this result supports the MST proposal that MI activates the same neural processes as actual execution. However, we also found that although both MI and actual execution significantly reduced STDT, there was a significant difference in the specific locations affected. Compared with MI, actual execution significantly reduced STDT in the index finger on the trained side. In contrast, MI significantly reduced STDT in the palm on the trained side, which was not observed with actual execution. This result further supports that the neural processes engaged by MI are influenced by high‐level cognitive processes; therefore, they are not entirely identical to those of actual execution. Conte et al. did not find a similar increase in STDT during MI as in motor execution [13], further demonstrating that the neural processes activated in MI differ from those in actual execution.

The phenomenon may be explained from the perspective of the performance and brain mechanisms in the learning process of motor imagery and actual execution. It is generally believed that although motor imagery has only an implicit “planning and simulation” stage and no explicit “execution” stage compared with actual execution, it still involves the activation of the motor action system to some extent [76], which also explains why it has an impact on somatosensory ability. Furthermore, research has found that compared with unskilled individuals, experts exhibit more structured characteristics during exercise [77]. Specifically, as proficiency increases, motor representation is developing in an increasingly refined direction, and this phenomenon is also observed in the comparison between actual execution and motor imagination. This phenomenon may explain why motor imagery and actual execution exhibit similar somatosensory changes, but show significant differences in kinematic results.

### 4.3 The Plasticity in Somatosensory‐Related Brain Regions Across Training

SEPs have been used to measure several important neurophysiological changes associated with different functions of the somatosensory system [65] and are involved in different stages of autonomic movement. They also play an important role in sensorimotor integration, and their plasticity may even predate the motor cortex in motor training [78]. Through the study of Parkinson's disease and dystonia, researchers generally believe that the expanded and overlapping receptive fields may be the cause of the decline in the temporal and spatial discrimination ability of these patients and are also characterized by abnormal changes in the primary somatosensory cortex [38, 62]. On the contrary, both actual execution and motor imagery have shown beneficial enhancements, both at the somatosensory discrimination and the cortical level. Many studies have demonstrated that the N20 peak and N20‐P25 components of SEPs are enhanced during and after motor training, both in actual execution [36, 79] and action observation [37]. Studies have also focused on changes in the spatial domain of SEPs, such as the correlation between movement deficits and impaired orientation sensitivity at the fingertips [38] and between the fingers [8] in dystonia patients. One study also compared the relationship between lateral inhibition and sensorimotor integration, suggesting that the integration pattern is strategically adjusted to the perceptual task, with differences being extracted faster in the comparison mode and consistency being extracted faster in the combination mode [80]. Our results show that, although actual motor training and MI do not change the temporal domain before execution, they enhance the plasticity of the spatial domain represented by SIR on the trained side, which is correlated with STDT changes, although there are differences across body locations. Considering that SIR shows a significant increase in patients with dystonia compared to healthy individuals [8], and that contiguous fingers are represented adjacently in S1 [81], we believe that training may have a special enhancement effect on this adjacent area. In addition, although SIR in the BRT group showed a correlation with motor scores on the trained side, considering the distribution of the data, the validity of this correlation still needs to be further explored. Nevertheless, our study supports that there are similarities in the main neural circuits and differences in specific neural processes between MI and actual execution in terms of somatosensory function, likely caused by the different cognitive processes involved in high‐level cognitive functions.

Finally, we collected the resting‐stage EEG data and performed a power spectral analysis on the assessment day. Our results show that actual execution and MI induce different plasticity changes. In actual execution, the plasticity of the gamma band in the bilateral parietal cortex and the theta band in the prefrontal cortex increased, and changes in the prefrontal cortex were significantly correlated with changes in STDT. In contrast, MI enhanced delta‐band plasticity in the ipsilateral parietal cortex on the trained side, which was significantly correlated with STDT changes. Previous studies have confirmed that neural oscillations in the gamma bands of the parietal cortex play a key role in motor control based on sensory feedback and are also related to motor training [82]. Ishii et al. found that, when using chopsticks, spectral power in the gamma band was significantly higher for unskilled users (such as Europeans) than for skilled users (such as Asians) [83]. Similarly, Hamada et al. found that the gamma enhancement in the parietal cortex occurred only when the movement was performed in accordance with the motor training [84]. Furthermore, the functions of the theta and gamma frequency bands in learning and their cross‐cortical coupling have also been taken into consideration. Studies have found that theta‐gamma phase‐amplitude coupling occurring in the hippocampus is regarded as an important learning‐related mechanism and is considered a cross‐cortical phenomenon [85]. Meanwhile, another study also found that the differences between complex training and simple training in different frequency of different cortices were enhanced, especially in the theta and gamma bands [86]. These studies all indicate that the coupling of the theta‐gamma frequency band may be the key to the training process of actual execution.

Although some studies have found widespread gamma enhancement during MI [87], our study did not bring about plastic changes, suggesting that gamma‐band enhancement in the parietal cortex may only be related to actual execution. Miladinovic et al. found that delta and theta band oscillations in the ipsilateral and contralateral parietal cortex on the trained side showed a significant decline after MI [88] which may be due to the rapid impact of MI on the sensorimotor cortex. Our study further found that this effect also manifests as enhanced plasticity of the delta band after long‐term MI training and that this enhancement was significantly correlated with STDT. Our findings are further supported by recent studies demonstrating that MI training for stroke patients is associated with spontaneous activity in the ipsilesional inferior parietal lobule [16, 89]. However, unexpectedly, plasticity enhancement in the prefrontal cortex was correlated with STDT after long‐term training. Although previous studies did not consider the prefrontal cortex to be a primary region involved in STDT encoding [4, 90], our observed correlation suggests that this hypothesis needs further examination.

Our study has some limitations. First, due to the sample size and the statistical analysis method, the statistical power may have been too low; therefore, some differences may have been overlooked. Second, due to the requirements of the experimental design, we compressed the time window for SEP measurement, which deprived us of the possibility of analyzing SEP components with longer latency periods. These should also be taken into account in subsequent studies. In addition, because of the limitations of experimental design and data collection, our study lacks depth in discussing the differences in neural mechanisms between MI and actual execution, especially the role of kinesthesia in motor imagery (such as dMI) needs to be further considered; therefore, more targeted experiments are needed to further discuss the differences between these techniques.

## 5 Conclusion

This study systematically compared the effects of actual execution, tactile control, MI, and no intervention on somatosensory temporal discrimination and kinematic performance by integrating EEG, SEP, and kinematic analyses. The key findings revealed that actual execution training (BRT) outperformed the other conditions in enhancing motor efficiency and reducing movement variability, particularly in index‐finger coordination. Both BRT and VGI improved STDT; however, based on divergent mechanisms, BRT targeted the index finger, while VGI influenced palm sensitivity. Neurophysiological data indicated that BRT enhanced gamma oscillations in the parietal regions and theta activity in the prefrontal cortex, whereas VGI modulated delta‐band power in the ipsilateral parietal cortex. SEP plasticity (SIR) correlated with STDT improvements, underscoring the role of primary somatosensory cortex reorganization. These results contribute to the understanding of motor training paradigms, demonstrating that execution and imagery engage overlapping but distinct neural circuits, with implications for rehabilitation strategies for neurological disorders.

## Author Contributions

Jinyan Zhang: Conceptualization, data curation, investigation, methodology, software, visualization, writing – original draft, writing – review and editing. Wangjun Zou: Data curation, visualization, writing – original draft. Binbin Gao: Data curation, investigation, writing – review and editing. Jinglong Wu: Methodology, resources, supervision. Zhilin Zhang: Investigation, methodology, resources, supervision, validation, writing – review and editing. Jian Zhang: Project administration, resources, supervision, validation, writing – review and editing. Luyao Wang: Project administration, resources, supervision, validation, writing – review and editing. Tianyi Yan: Project administration, resources, supervision, validation, writing – review and editing.

## Ethics Statement

The experiment was conducted with the understanding and written informed consent of each participant, conforming to the Declaration of Helsinki and the standards established by the Institutional Review Board and Ethics Committee of the Beijing Institute of Technology (Approval no BIT‐EC‐H‐2023183). The experimental protocol was approved by the Institutional Review Board and Ethics Committee of the Beijing Institute of Technology and carried out in accordance with relevant guidelines and regulations.

## Conflicts of Interest

The authors declare no conflicts of interest.

## Supporting information


**Appendix S1:** cns70564‐sup‐0001‐AppendixS1.docx.

## Data Availability

The data that support the findings of this study are available from the corresponding author, Zhilin Zhang, upon reasonable request.
