# Supplementary material for: Regulation of Somatosensory Temporal Discrimination Threshold Through Motor Training: An EEG and Kinematics Study
Source: CNS Neurosci Ther. 2025 Aug 26;31(8):e70564. doi: 10.1111/cns.70564 (PMC12378431; doi:10.1111/cns.70564)
Supplement: Supplementary file 1 — Appendix S1: cns70564‐sup‐0001‐AppendixS1.docx. [file CNS-31-e70564-s001.docx]

Table S1. Statistical comparison of demographics

|  | | BRT (n = 12) | Tactile (n = 12) | VGI (n = 12) | Control (n = 12) | Statistical scores |
| --- | --- | --- | --- | --- | --- | --- |
| **Age (years)** | | 23.00 | 24.00 | 23.00 | 23.00 | χ² = 3.246 |
|  |  | [22.06, 25.10] | [23.23, 25.27] | [22.27, 24.39] | [22.35, 24.48] | (p = 0.355) |
| **Gender** | Women | 6 | 5 | 6 | 6 | Chi-square = 0.250 |
|  | Men | 6 | 7 | 6 | 6 | (p=0.969) |
| **Hand length (cm)** | | 18.55 | 18.25 | 18.00 | 18.00 | χ² = 4.814 |
|  |  | [18.15, 18.69] | [17.80 19.02] | [17.46, 18.57] | [17.48, 18.70] | (p = 0.186) |
| **Palm width (cm)** | | 10.55 | 10.70 | 10.20 | 9.85 | χ² = 3.235 |
|  |  | [10.19, 11.61] | [10.14, 11.45] | [9.82, 11.21] | [9.56 11.01] | (p = 0.357) |
| **KVIQ** | Vividness | 41.50 | 47.00 | 48.00 | 42.00 | χ² = 3.623 |
|  |  | [33.38, 46.96] | [42.48, 48.52] | [43.15, 49.18] | [37.18, 45.82] | (p = 0.305) |
|  | Kinaesthetic | 39.00 | 41.50 | 43.50 | 35.00 | χ² = 5.082 |
|  |  | [34.15, 43.85] | [36.70, 46.30] | [41.10, 46.39] | [30.22, 42.44] | (p = 0.166) |
| **EIH** |  | 88.85 | 77.78 | 86.19 | 83.75 | χ² = 3.117 |
|  |  | [79.22, 96.14] | [69.46, 85.04] | [65.87, 90.34] | [54.00, 91.16] | (p = 0.374) |

Values are mean [95% confidence interval, CI].

KVIQ = Kinaesthetic Vividness Inventory Questionnaire; EIH = Edinburgh Handedness Inventory.


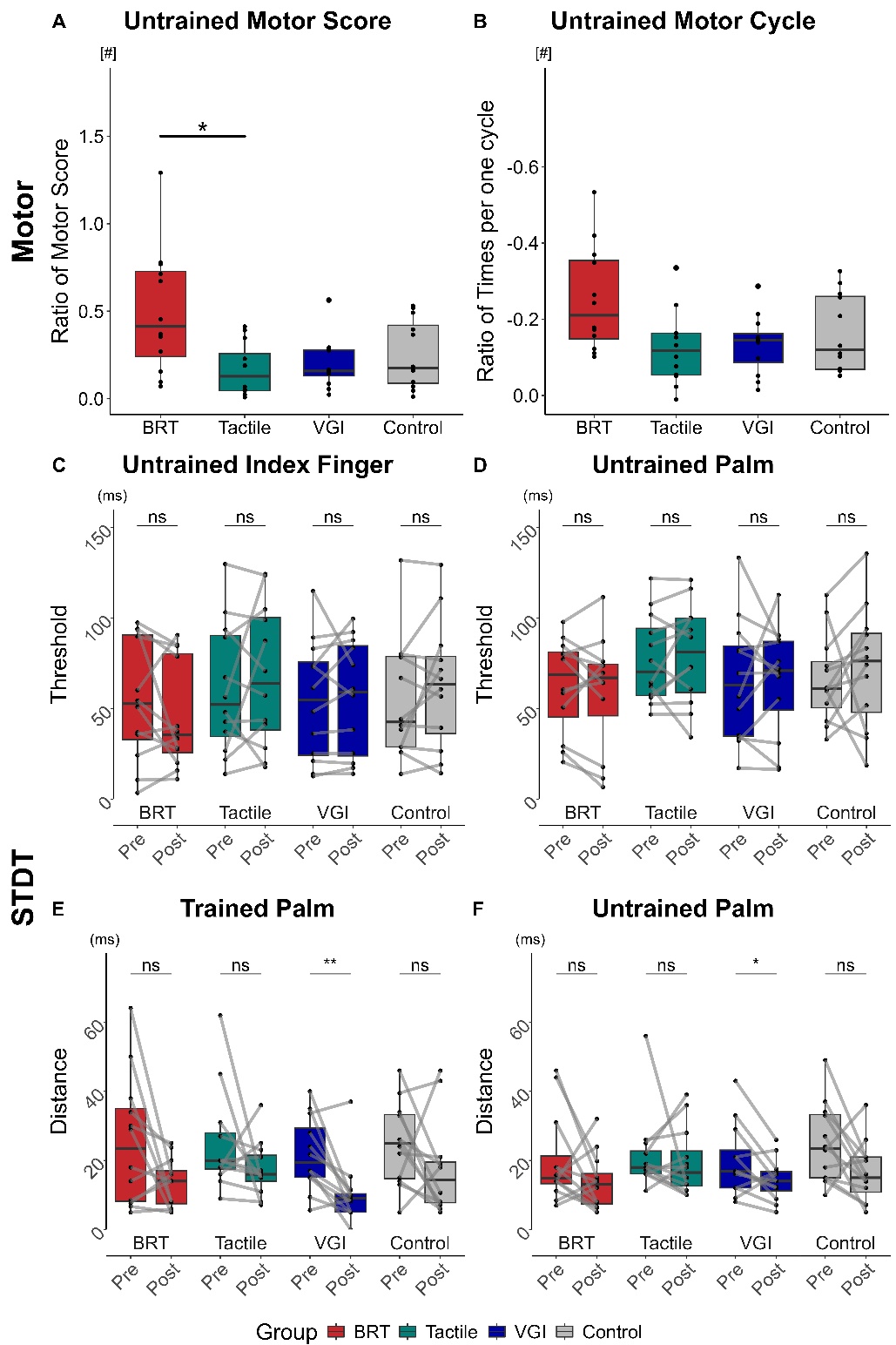


Figure S1. Effects of training technique (BRT, Tactile, VGI, Control) on motor performance, and threshold on untrained hand and distance on each hand of STDT. (A-B) The box plot shows the ratios of motor scores and motor cycle (normalized to baseline) in the BRT, tactile, VGI, and Control on untrained hand. (C - D) The box plot compared pre- and post-training of STDT at the index finger and palm in each group on untrained hand. (E - F) The box plot compared pre- and post-training of the distance of STDT at the index finger and palm in each group on untrained hand. (*: p < 0.05, **: p < 0.01, ***: p < 0.001, ns: p > 0.05).

Table S2. Statistical comparison of motor score and motor cycle

|  |  | **Value** |  |  | **P (r)** | |  |  |
| --- | --- | --- | --- | --- | --- | --- | --- | --- |
|  | Group | Pre | Post | Delta | Pre-Post | to Tactile | to VGI | to Control |
| **Motor Score (N)** | |  |  |  |  |  |  |  |
|  | **Trained Hand** |  |  |  |  |  |  |  |
|  | BRT | 17.40 | 32.76 | 0.83 | **<0.001** | **<0.001** | **<0.001** | **0.004** |
|  |  | [13.31, 20.90] | [30.00, 36.12] | [0.61, 1.07] | **(0.88)** | **(0.82)** | **(0.81)** | **(0.64)** |
|  | Tactile | 20.45 | 23.20 | 0.21 | **0.005** |  | 1 | 1 |
|  |  | [15.10, 24.55] | [18.71, 30.32] | [0.10, 0.25] | **(0.82)** |  | (0.02) | (0.15) |
|  | VGI | 24.10 | 27.70 | 0.21 | **<0.001** |  |  | 1 |
|  |  | [20.40, 27.70] | [24.60, 32.30] | [0.10, 0.22] | **(0.86)** |  |  | (0.18) |
|  | Control | 12.87 | 15.70 | 0.21 | **<0.001** |  |  |  |
|  |  | [9.82, 19.97] | [13.64, 23.40] | [0.17, 0.34] | **(0.88)** |  |  |  |
|  | **Untrained Hand** |  |  |  |  |  |  |  |
|  | BRT | 16.06 | 23.10 | 0.41 | **<0.001** | **0.033** | 0.121 | 0.355 |
|  |  | [10.39, 18.20] | [17.60, 25.20] | [0.21, 0.74] | **(0.88)** | **(0.55)** | (0.46) | (0.35) |
|  | Tactile | 19.50 | 21.45 | 0.13 | **0.003** |  | 1 | 0.957 |
|  |  | [13.97, 24.43] | [18.10, 27.44] | [0.05, 0.29] | **(0.88)** |  | (0.12) | (0.21) |
|  | VGI | 19.21 | 22.20 | 0.16 | **0.003** |  |  | 1 |
|  |  | [17.58, 21.56] | [21.58, 24.80] | [0.12, 0.28] | **(0.88)** |  |  | (0.14) |
|  | Control | 14.18 | 19.40 | 0.17 | **<0.001** |  |  |  |
|  |  | [12.94, 22.28] | [15.90, 24.87] | [0.08, 0.44] | **(0.88)** |  |  |  |
| **Motor Cycle (s)** | |  |  |  |  |  |  |  |
|  | **Trained Hand** |  |  |  |  |  |  |  |
|  | BRT | 1.02 | 0.64 | -0.37 | **<0.001** | **<0.001** | **<0.001** | **0.022** |
|  |  | [0.96, 1.18] | [0.59, 0.67] | [-0.45, -0.34] | **(0.88)** | **(0.75)** | **(0.75)** | **(0.54)** |
|  | Tactile | 0.95 | 0.90 | -0.16 | **<0.001** |  | 0.81 | 0.64 |
|  |  | [0.78, 1.16] | [0.70, 0.93] | [-0.21, -0.06] | **(0.88)** |  | (0.05) | (0.21) |
|  | VGI | 0.80 | 0.74 | -0.12 | **<0.001** |  |  | 0.156 |
|  |  | [0.71, 0.92] | [0.62, 0.78] | [-0.16, -0.08] | **(0.88)** |  |  | (0.40) |
|  | Control | 1.31 | 1.08 | -0.16 | **<0.001** |  |  |  |
|  |  | [0.99, 1.87] | [0.83, 1.29] | [-0.26, -0.14] | **(0.88)** |  |  |  |
|  | **Untrained Hand** |  |  |  |  |  |  |  |
|  | BRT | 1.10 | 0.81 | -0.24 | **<0.001** | 0.052 | 0.094 | 0.209 |
|  |  | [0.99, 1.59] | [0.74, 1.04] | [-0.35, -0.16] | **(0.88)** | (0.51) | (0.47) | (0.39) |
|  | Tactile | 0.97 | 0.85 | -0.13 | **<0.001** |  | 1 | 1 |
|  |  | [0.79, 1.24] | [0.68, 0.95] | [-0.16, -0.05] | **(0.86)** |  | (0.03) | (0.14) |
|  | VGI | 0.97 | 0.83 | -0.15 | **<0.001** |  |  | 1 |
|  |  | [0.84, 1.00] | [0.73, 0.86] | [-0.17, -0.07] | **(0.86)** |  |  | (0.08) |
|  | Control | 1.17 | 0.95 | -0.12 | **<0.001** |  |  |  |
|  |  | [0.84, 1.33] | [0.76, 1.14] | [-0.26, -0.07] | (0.88) |  |  |  |

Values are median [95% confidence interval, CI], calculated by Bootstrap resample method. Random seed was set to 123 for reproducibility.

r means Rank-Biserial Correlation.

BRT = ball-rotation training, for actual execution.

Tactile = tactile control, for simple gripping.

VGI = visual-guided imagery, for motor imagery, MI.

Control for baseline conditions without intervention.

Table S3. Statistical comparison of STDT

|  |  | **BRT** |  | **P** | **Tactile** |  | **P** | **VGI** |  | **P** | **Control** |  | **P** |
| --- | --- | --- | --- | --- | --- | --- | --- | --- | --- | --- | --- | --- | --- |
| **Location** | | Pre | Post | (r) | Pre | Post | (r) | Pre | Post | (r) | Pre | Post | (r) |
| **Threshold (ms)** | | |  |  |  |  |  |  |  |  |  |  |  |
|  | **Trained Hand** | |  |  |  |  |  |  |  |  |  |  |  |
|  | Index | 64.02 | 43.75 | **0.007** | 49.49 | 49.68 | 0.519 | 69.57 | 59.38 | **0.034** | 56.43 | 69.98 | 0.733 |
|  |  | [29.83, 90.34] | [19.62, 74.74] | **(0.75)** | [35.86, 78.58] | [26.75, 75.69] | (0.20) | [27.37, 101.84] | [17.98, 85.47] | **(0.61)** | [40.59, 96.66] | [48.42, 83.16] | (0.11) |
|  | Thumb | 62.67 | 56.93 | 0.176 | 78.05 | 74.29 | 0.677 | 63.77 | 75.56 | 0.47 | 65.55 | 71.23 | 0.97 |
|  |  | [18.71, 111.35] | [27.09, 88.02] | (0.40) | [35.45, 104.17] | [41.10, 108.13] | (0.13) | [30.95, 93.25] | [20.28, 86.77] | (0.23) | [54.07, 94.61] | [58.30, 90.90] | (0.02) |
|  | Little | 44.5 | 48.23 | 0.91 | 38.91 | 53.8 | 0.47 | 66 | 56.15 | 0.339 | 41.29 | 48.68 | 0.339 |
|  |  | [20.07, 82.62] | [22.81, 79.70] | (0.04) | [32.36, 88.54] | [27.09, 103.61] | (0.23) | [22.81, 85.81] | [19.43, 76.33] | (0.29) | [30.84, 72.91] | [39.59, 87.82] | (0.29) |
|  | Palm | 77.55 | 63.29 | 0.266 | 89.56 | 68.33 | 0.204 | 73.43 | 61.13 | **0.003** | 83.46 | 76.16 | 0.266 |
|  |  | [36.76, 93.53] | [40.71, 80.74] | (0.34) | [43.90, 92.55] | [50.68, 85.52] | (0.38) | [44.70, 99.32] | [29.59, 76.08] | **(0.79)** | [48.69, 89.52] | [53.03, 86.99] | (0.34) |
|  | Wrist | 96.11 | 81.37 | 0.38 | 83.01 | 81.17 | 0.569 | 85.29 | 78.74 | 0.569 | 73.78 | 82.41 | 0.064 |
|  |  | [47.26, 113.39] | [68.32, 93.38] | (0.27) | [66.99, 103.61] | [57.24, 98.31] | (0.18) | [48.24, 94.26] | [43.85, 94.64] | (0.18) | [62.60, 95.74] | [69.30, 103.39] | (0.54) |
|  | **Untrained Hand** | |  |  |  |  |  |  |  |  |  |  |  |
|  | Index | 52.64 | 35.41 | 0.301 | 52.16 | 64.02 | 0.85 | 54.84 | 59.09 | 0.266 | 42.54 | 63.55 | 0.569 |
|  |  | [29.97, 91.69] | [23.82, 81.67] | (0.32) | [31.99, 91.45] | [34.81, 101.80] | (0.07) | [23.09, 78.19] | [22.47, 85.69] | (0.34) | [28.83, 79.02] | [32.95, 80.74] | (0.18) |
|  | Thumb | 63.24 | 55.09 | 0.47 | 81.9 | 68.58 | 0.233 | 81.49 | 53.74 | 0.129 | 65.22 | 67.07 | 0.47 |
|  |  | [20.23, 78.87] | [26.69, 82.35] | (0.23) | [28.87, 100.89] | [25.16, 97.07] | (0.36) | [21.55, 103.81] | [19.68, 93.02] | (0.45) | [28.56, 81.66] | [42.53, 84.30] | (0.23) |
|  | Little | 56.02 | 45.6 | 0.569 | 56.98 | 62.57 | 0.233 | 76.01 | 55.22 | 0.85 | 64.05 | 69.07 | 0.38 |
|  |  | [33.06, 78.30] | [25.64, 84.74] | (0.18) | [34.46, 93.82] | [33.19, 87.24] | (0.36) | [27.87, 79.49] | [32.75, 83.99] | (0.07) | [35.93, 84.12] | [51.89, 91.07] | (0.27) |
|  | Palm | 68.81 | 67.01 | 0.47 | 70.36 | 81.16 | 0.519 | 63.04 | 70.89 | 0.791 | 60.96 | 76.19 | 0.339 |
|  |  | [39.89, 82.36] | [36.61, 75.05] | (0.23) | [56.87, 96.79] | [57.00, 100.03] | (0.20) | [34.33, 86.86] | [43.03, 87.42] | (0.09) | [47.95, 78.39] | [44.06, 92.29] | (0.29) |
|  | Wrist | 90.49 | 78.5 | 0.677 | 88.98 | 86 | 0.85 | 77.16 | 76.26 | 0.424 | 75.35 | 75.54 | 0.47 |
|  |  | [49.78, 95.98] | [60.47, 84.71] | (0.13) | [67.90, 104.29] | [68.25, 107.07] | (0.07) | [45.67, 100.94] | [57.36, 101.06] | (0.25) | [64.41, 95.25] | [57.50, 93.15] | (0.23) |
| **Distance (ms)** | | |  |  |  |  |  |  |  |  |  |  |  |
|  | **Trained Hand** | |  |  |  |  |  |  |  |  |  |  |  |
|  | Index | 16.43 | 15 | 0.176 | 22.5 | 20.5 | 0.791 | 21.53 | 11.5 | 0.204 | 18 | 14 | 0.052 |
|  |  | [12.00, 37.00] | [10.89, 24.50] | (0.41) | [18.50, 26.07] | [14.21, 28.50] | (0.09) | [11.50, 27.14] | [6.85, 17.50] | (0.38) | [10.47, 45.50] | [10.50, 22.50] | (0.57) |
|  | Thumb | 18.5 | 19 | 0.85 | 16.84 | 20.5 | 0.791 | 17.5 | 11.81 | 0.233 | 21.76 | 17.68 | 0.176 |
|  |  | [10.32, 22.45] | [9.27, 28.00] | (0.07) | [12.31, 33.00] | [10.17, 35.50] | (0.09) | [10.31, 23.02] | [8.71, 17.13] | (0.36) | [13.70, 38.00] | [14.76, 20.00] | (0.41) |
|  | Little | 16.59 | 9.5 | 0.97 | 12.79 | 14.33 | 0.233 | 16.35 | 12.04 | 0.47 | 16.5 | 14 | 0.519 |
|  |  | [8.88, 23.48] | [7.58, 25.82] | ((0.02) | [7.87, 21.50] | [10.92, 21.50] | (0.36) | [9.85, 20.29] | [6.87, 20.00] | (0.23) | [13.00, 23.30] | [10.50, 23.00] | (0.20) |
|  | Palm | 23.5 | 14 | 0.129 | 19.96 | 16 | 0.151 | 19.36 | 9.03 | **0.002** | 25 | 14.32 | 0.151 |
|  |  | [8.03, 36.00] | [6.96, 18.00] | (0.46) | [17.00, 29.00] | [13.00, 22.00] | (0.43) | [15.05, 30.82] | [5.00, 10.75] | **(0.81)** | [14.50, 33.50] | [7.54, 20.00] | (0.43) |
|  | Wrist | 17.5 | 15.45 | 0.38 | 19 | 16.69 | 0.97 | 15.7 | 14 | 0.151 | 23 | 13.5 | 0.266 |
|  |  | [11.84, 22.00] | [11.59, 23.00] | (0.27) | [13.00, 28.00] | [9.84, 32.00] | (0.02) | [12.50, 26.00] | [11.00, 19.50] | (0.43) | [14.50, 35.00] | [11.00, 22.50] | (0.34) |
|  | **Untrained Hand** | |  |  |  |  |  |  |  |  |  |  |  |
|  | Index | 24 | 19 | 0.064 | 20.21 | 17.71 | 0.47 | 12.5 | 19.04 | 0.569 | 14.5 | 20.98 | 0.519 |
|  |  | [17.72, 34.00] | [9.52, 26.73] | (0.54) | [11.77, 26.50] | [12.50, 21.74] | (0.22) | [7.85, 26.00] | [12.44, 24.50] | (0.18) | [9.64, 30.00] | [8.13, 26.00] | (0.20) |
|  | Thumb | 14.79 | 17.14 | 0.519 | 22.5 | 18.5 | 1 | 15.51 | 12 | 0.791 | 11 | 19.5 | 0.092 |
|  |  | [9.12, 22.50] | [12.21, 25.00] | (0.20) | [12.80, 39.50] | [15.68, 27.77] | 0 | [9.27, 31.00] | [8.39, 27.89] | (0.09) | [6.92, 16.75] | [14.50, 25.50] | (0.50) |
|  | Little | 12.96 | 17 | 0.733 | 19.5 | 19 | 1 | 20 | 20 | 0.791 | 14.5 | 17.5 | 0.791 |
|  |  | [10.39, 46.00] | [6.23, 22.50] | (0.11) | [11.00, 28.00] | [14.50, 25.00] | 0 | [11.34, 32.20] | [9.87, 25.50] | (0.09) | [10.79, 22.00] | [12.00, 24.50] | (0.09) |
|  | Palm | 14.9 | 13.08 | 0.204 | 18 | 16.5 | 0.97 | 16.83 | 14 | **0.043** | 23.5 | 15 | 0.064 |
|  |  | [12.56, 24.50] | [7.15, 17.39] | (0.38) | [16.07, 23.50] | [12.17, 24.50] | (0.02) | [12.03, 25.00] | [10.45, 17.03] | **(0.59)** | [14.95, 33.50] | [9.60, 20.99] | (0.54) |
|  | Wrist | 18.5 | 14 | 0.38 | 21.5 | 16 | 0.85 | 14.5 | 18 | 0.092 | 16.5 | 13.72 | 0.266 |
|  |  | [10.50, 25.81] | [11.80, 22.50] | (0.27) | [16.24, 26.00] | [13.50, 30.00] | ((0.07) | [11.36, 17.21] | [15.00, 25.00] | (0.50) | [12.97, 23.50] | [10.37, 22.50] | (0.34) |

Values are median [95% confidence interval, CI], calculated by Bootstrap resample method. Random seed was set to 123 for reproducibility.

r means Rank-Biserial Correlation.

BRT = ball-rotation training, for actual execution.

Tactile = tactile control, for simple gripping.

VGI = visual-guided imagery, for motor imagery, MI.

Control for baseline conditions without intervention.


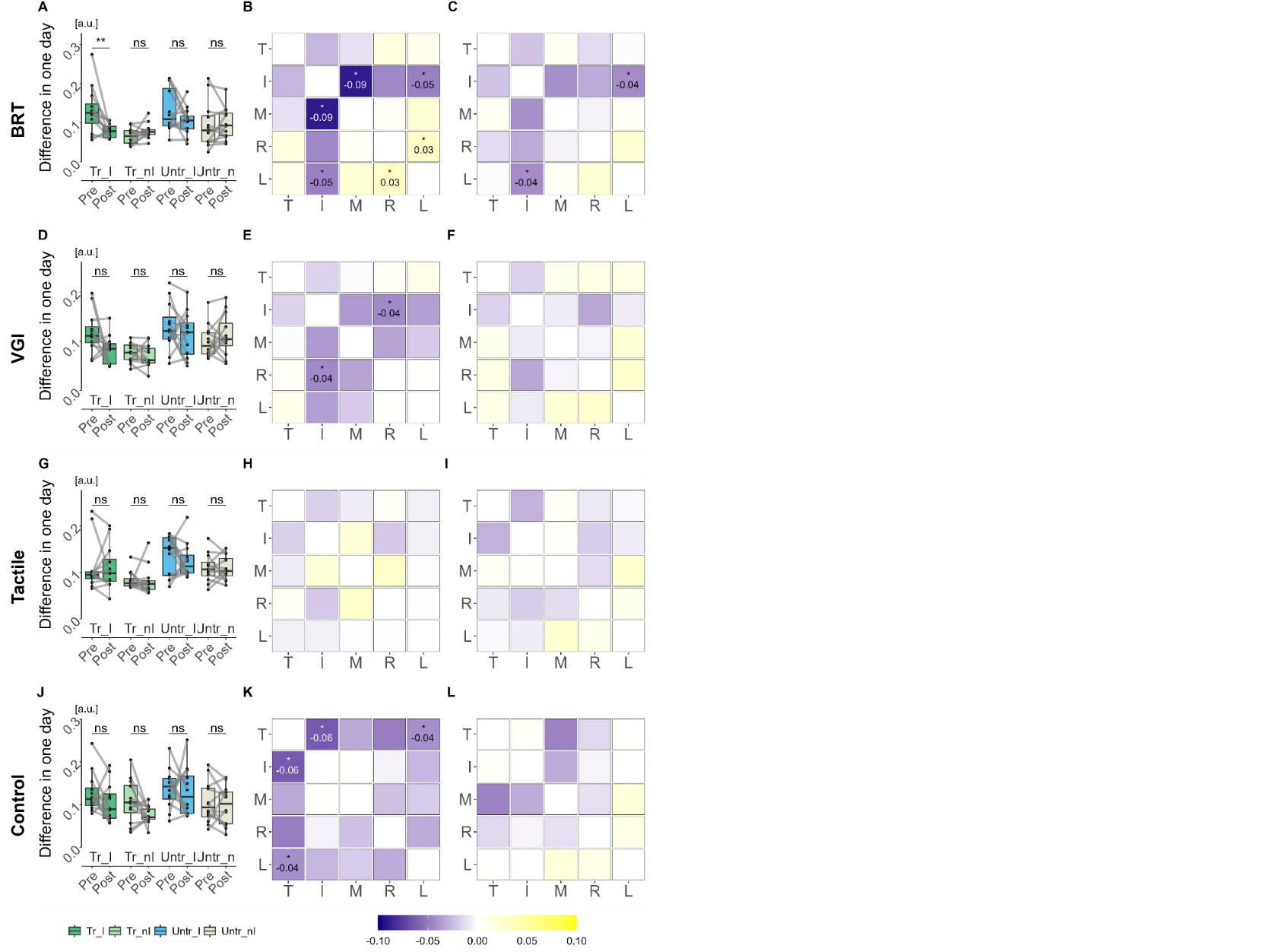


Figure S2. Difference in digit coordination patterns on total and each hand. (A, D, G, J) The box plot compared pre- and post-training of the overall digit coordination patterns across hand based on index finger in each group. The connecting lines show individual group trends. (B, C, E, F, H, I, K, L) Difference in digit coordination patterns. combination of digits across assessment days. T = Thumb finger, I = Index finger, M = middle finger, R = Ring finger, L = Little finger. Zero indicates a coordination pattern that is identical across two assessment days. (*: p < 0.05, **: p < 0.01, ***: p < 0.001, ns: p > 0.05).

Table S4. Statistical comparison of the overall digit coordination patterns

|  | **BRT** |  | **P** | **Tactile** |  | **P** | **VGI** |  | **P** | **Control** |  | **P** |
| --- | --- | --- | --- | --- | --- | --- | --- | --- | --- | --- | --- | --- |
| **Location** | Pre | Post | (r) | Pre | Post | (r) | Pre | Post | (r) | Pre | Post | (r) |
| **Trained Index** | 0.13 | 0.08 | **0.005** | 0.1 | 0.1 | 0.685 | 0.11 | 0.08 | 0.092 | 0.11 | 0.09 | 0.204 |
|  | [0.11, 0.16] | [0.06, 0.09] | **(0.75)** | [0.09, 0.10] | [0.08, 0.12] | (0.13) | [0.09, 0.13] | [0.05, 0.10] | (0.50) | [0.10, 0.14] | [0.07, 0.13] | ((0.39) |
| **Trained nonIndex** | 0.06 | 0.08 | 0.191 | 0.07 | 0.08 | 0.685 | 0.08 | 0.06 | 0.11 | 0.11 | 0.07 | 0.11 |
|  | [0.05, 0.08] | [0.07, 0.08] | (0.38) | [0.07, 0.08] | [0.06, 0.09] | (0.13) | [0.06, 0.09] | [0.05, 0.09] | (0.48) | [0.07, 0.15] | [0.07, 0.09] | (0.48) |
| **Untrained Index** | 0.12 | 0.11 | 0.127 | 0.15 | 0.1 | 0.497 | 0.12 | 0.12 | 0.301 | 0.14 | 0.12 | 0.424 |
|  | [0.09, 0.21] | [0.09, 0.13] | (0.44) | [0.08, 0.17] | [0.10, 0.13] | (0.20) | [0.10, 0.16] | [0.07, 0.14] | (0.32) | [0.11, 0.16] | [0.08, 0.17] | (0.25) |
| **Untrained nonIndex** | 0.09 | 0.1 | 0.946 | 0.11 | 0.1 | 0.839 | 0.09 | 0.1 | 0.301 | 0.09 | 0.1 | 0.677 |
|  | [0.05, 0.13] | [0.07, 0.13] | (0.03) | [0.08, 0.12] | [0.09, 0.13] | (0.07) | [0.07, 0.12] | [0.09, 0.14] | (0.32) | [0.07, 0.14] | [0.05, 0.13] | (0.13) |

Values are median [95% confidence interval, CI], calculated by Bootstrap resample method. Random seed was set to 123 for reproducibility.

r means Rank-Biserial Correlation.

BRT = ball-rotation training, for actual execution.

Tactile = tactile control, for simple gripping.

VGI = visual-guided imagery, for motor imagery, MI.

Control for baseline conditions without intervention.

Trained/Untrained: the hand had (or had not) been trained on training day.

Index/nonIndex: the total of digit coordination patterns including (or not) index finger.

Table S5. Statistical comparison of each digit coordination pattern

|  |  | **BRT** |  | **P** | **Tactile** |  | **P** | **VGI** |  | **P** | **Control** |  | **P** |
| --- | --- | --- | --- | --- | --- | --- | --- | --- | --- | --- | --- | --- | --- |
| Combinations | | Pre | Post | (r) | Pre | Post | (r) | Pre | Post | (r) | Pre | Post | (r) |
|  | **Trained** |  |  |  |  |  |  |  |  |  |  |  |  |
|  | T_I | 0.08 | 0.08 | 0.497 | 0.11 | 0.09 | 0.946 | 0.1 | 0.07 | 0.266 | 0.15 | 0.08 | **0.043** |
|  |  | [0.07, 0.14] | [0.07, 0.09] | (0.20) | [0.07, 0.14] | [0.06, 0.12] | (0.03) | [0.07, 0.12] | [0.04, 0.14] | (0.34) | [0.09, 0.19] | [0.04, 0.11] | **(0.59)** |
|  | *T_M* | 0.08 | 0.09 | 0.635 | 0.14 | 0.11 | 0.542 | 0.1 | 0.1 | 0.91 | 0.14 | 0.1 | 0.339 |
|  |  | [0.07, 0.14] | [0.08, 0.12] | (0.15) | [0.11, 0.16] | [0.10, 0.14] | (0.18) | [0.07, 0.12] | [0.07, 0.12] | (0.04) | [0.07, 0.19] | [0.09, 0.13] | (0.29) |
|  | *T_R* | 0.07 | 0.07 | 0.244 | 0.1 | 0.08 | 0.839 | 0.09 | 0.08 | 0.733 | 0.17 | 0.1 | 0.077 |
|  |  | [0.05, 0.08] | [0.06, 0.11] | (0.34) | [0.06, 0.11] | [0.07, 0.09] | (0.07) | [0.07, 0.10] | [0.07, 0.11] | (0.11) | [0.09, 0.19] | [0.06, 0.15] | (0.52) |
|  | *T_L* | 0.07 | 0.08 | 0.376 | 0.08 | 0.07 | 0.893 | 0.06 | 0.08 | 0.339 | 0.13 | 0.07 | **0.034** |
|  |  | [0.04, 0.09] | [0.06, 0.09] | (0.26) | [0.05, 0.09] | [0.05, 0.08] | (0.05) | [0.05, 0.09] | [0.06, 0.10] | (0.29) | [0.09, 0.16] | [0.05, 0.13] | **(0.61)** |
|  | I_M | 0.18 | 0.08 | **0.001** | 0.11 | 0.14 | 0.542 | 0.13 | 0.09 | 0.092 | 0.12 | 0.1 | 0.85 |
|  |  | [0.09, 0.20] | [0.06, 0.09] | **(0.82)** | [0.07, 0.13] | [0.11, 0.15] | (0.18) | [0.09, 0.18] | [0.07, 0.13] | (0.50) | [0.10, 0.15] | [0.07, 0.19] | (0.07) |
|  | I_R | 0.11 | 0.09 | 0.127 | 0.09 | 0.09 | 0.588 | 0.1 | 0.06 | **0.034** | 0.08 | 0.09 | 0.85 |
|  |  | [0.08, 0.19] | [0.07, 0.10] | (0.44) | [0.06, 0.13] | [0.06, 0.11] | (0.16) | [0.09, 0.13] | [0.05, 0.08] | **(0.61)** | [0.06, 0.16] | [0.05, 0.17] | (0.07) |
|  | I_L | 0.1 | 0.08 | **0.04** | 0.09 | 0.08 | 0.839 | 0.1 | 0.08 | 0.233 | 0.11 | 0.09 | 0.077 |
|  |  | [0.08, 0.14] | [0.05, 0.10] | **(0.57)** | [0.07, 0.12] | [0.07, 0.10] | (0.07) | [0.08, 0.12] | [0.05, 0.10] | (0.36) | [0.09, 0.15] | [0.07, 0.11] | (0.52) |
|  | *M_R* | 0.06 | 0.06 | 0.946 | 0.06 | 0.06 | 0.11 | 0.07 | 0.04 | 0.064 | 0.07 | 0.04 | 0.266 |
|  |  | [0.03, 0.06] | [0.04, 0.07] | (0.03) | [0.04, 0.07] | [0.05, 0.10] | (0.45) | [0.05, 0.11] | [0.02, 0.06] | (0.54) | [0.05, 0.09] | [0.04, 0.07] | (0.34) |
|  | *M_L* | 0.06 | 0.07 | 0.244 | 0.09 | 0.08 | 0.685 | 0.07 | 0.07 | 0.11 | 0.07 | 0.06 | 0.97 |
|  |  | [0.05, 0.07] | [0.05, 0.12] | (0.34) | [0.07, 0.10] | [0.06, 0.10] | (0.13) | [0.05, 0.11] | [0.04, 0.09] | (0.48) | [0.05, 0.10] | [0.05, 0.07] | (0.02) |
|  | *R_L* | 0.02 | 0.05 | **0.005** | 0.04 | 0.02 | 0.946 | 0.03 | 0.03 | 0.733 | 0.04 | 0.03 | 0.092 |
|  |  | [0.02, 0.03] | [0.03, 0.07] | **(0.75)** | [0.03, 0.05] | [0.02, 0.08] | (0.03) | [0.01, 0.03] | [0.02, 0.05] | (0.11) | [0.02, 0.13] | [0.02, 0.04] | (0.50) |
|  | **Untrained** |  |  |  |  |  |  |  |  |  |  |  |  |
|  | T_I | 0.1 | 0.1 | 0.542 | 0.15 | 0.1 | 0.414 | 0.09 | 0.07 | 0.092 | 0.09 | 0.08 | 0.97 |
|  |  | [0.09, 0.16] | [0.07, 0.12] | (0.18) | [0.07, 0.20] | [0.07, 0.14] | (0.24) | [0.06, 0.11] | [0.05, 0.10] | (0.50) | [0.07, 0.11] | [0.05, 0.14] | (0.02) |
|  | *T_M* | 0.09 | 0.1 | 0.588 | 0.09 | 0.09 | 0.893 | 0.08 | 0.1 | 0.85 | 0.11 | 0.08 | 0.151 |
|  |  | [0.07, 0.11] | [0.08, 0.11] | (0.16) | [0.08, 0.11] | [0.08, 0.14] | (0.05) | [0.07, 0.15] | [0.08, 0.16] | (0.07) | [0.06, 0.18] | [0.06, 0.10] | (0.43) |
|  | *T_R* | 0.08 | 0.08 | 0.735 | 0.07 | 0.07 | 0.685 | 0.06 | 0.08 | 0.233 | 0.08 | 0.06 | 0.233 |
|  |  | [0.05, 0.10] | [0.07, 0.08] | (0.11) | [0.06, 0.12] | [0.05, 0.10] | (0.13) | [0.05, 0.09] | [0.06, 0.10] | (0.36) | [0.06, 0.13] | [0.05, 0.09] | (0.36) |
|  | *T_L* | 0.07 | 0.07 | 0.542 | 0.08 | 0.11 | 0.839 | 0.07 | 0.06 | 0.791 | 0.09 | 0.08 | 0.85 |
|  |  | [0.05, 0.13] | [0.05, 0.12] | (0.18) | [0.07, 0.14] | [0.08, 0.13] | (0.68) | [0.04, 0.10] | [0.04, 0.12] | (0.09) | [0.05, 0.11] | [0.06, 0.14] | (0.07) |
|  | I_M | 0.11 | 0.1 | 0.191 | 0.14 | 0.12 | 0.893 | 0.11 | 0.14 | 0.97 | 0.18 | 0.14 | 0.176 |
|  |  | [0.08, 0.20] | [0.06, 0.16] | (0.38) | [0.09, 0.16] | [0.08, 0.18] | (0.05) | [0.09, 0.20] | [0.11, 0.17] | (0.02) | [0.09, 0.24] | [0.09, 0.16] | (0.41) |
|  | I_R | 0.11 | 0.12 | 0.216 | 0.1 | 0.1 | 0.542 | 0.11 | 0.08 | 0.424 | 0.14 | 0.12 | 0.91 |
|  |  | [0.08, 0.23] | [0.09, 0.14] | (0.36) | [0.08, 0.18] | [0.07, 0.13] | (0.18) | [0.09, 0.22] | [0.06, 0.15] | (0.25) | [0.11, 0.19] | [0.07, 0.20] | (0.04) |
|  | I_L | 0.13 | 0.08 | **0.048** | 0.13 | 0.11 | 0.735 | 0.15 | 0.13 | 1 | 0.14 | 0.13 | 0.91 |
|  |  | [0.09, 0.19] | [0.06, 0.11] | **(0.55)** | [0.07, 0.17] | [0.09, 0.15] | (0.11) | [0.11, 0.16] | [0.07, 0.17] | (0) | [0.12, 0.14] | [0.09, 0.17] | (0.04) |
|  | *M_R* | 0.05 | 0.06 | 0.588 | 0.09 | 0.09 | 0.305 | 0.07 | 0.07 | 0.622 | 0.07 | 0.06 | 0.733 |
|  |  | [0.04, 0.08] | [0.04, 0.07] | (0.16) | [0.06, 0.17] | [0.08, 0.11] | (0.30) | [0.05, 0.10] | [0.05, 0.10] | (0.16) | [0.04, 0.11] | [0.04, 0.10] | (0.11) |
|  | *M_L* | 0.08 | 0.13 | 0.685 | 0.11 | 0.13 | 0.216 | 0.12 | 0.15 | 0.47 | 0.11 | 0.12 | 0.677 |
|  |  | [0.05, 0.13] | [0.08, 0.16] | (0.13) | [0.08, 0.14] | [0.10, 0.17] | (0.36) | [0.10, 0.19] | [0.10, 0.20] | (0.23) | [0.07, 0.16] | [0.06, 0.21] | (0.13) |
|  | *R_L* | 0.09 | 0.15 | 0.376 | 0.11 | 0.13 | 0.635 | 0.12 | 0.15 | 0.47 | 0.1 | 0.08 | 0.47 |
|  |  | [0.02, 0.17] | [0.06, 0.21] | (0.26) | [0.07, 0.19] | [0.06, 0.17] | (0.15) | [0.05, 0.17] | [0.10, 0.20] | (0.23) | [0.04, 0.18] | [0.05, 0.23] | (0.23) |

Values are median [95% confidence interval, CI], calculated by Bootstrap resample method. Random seed was set to 123 for reproducibility.

r means Rank-Biserial Correlation.

BRT = ball-rotation training, for actual execution.

Tactile = tactile control, for simple gripping.

VGI = visual-guided imagery, for motor imagery, MI.

Control for baseline conditions without intervention.

Trained/Untrained: the hand had (or had not) been trained on training day.

T = Thumb finger; I = Index finger; M = Middle finger; R = Ring finger; L = Little finger.

Table S6. Statistical comparison of SEP

|  |  | **BRT** |  | **P** | **Tactile** |  | **P** | **VGI** |  | **P** | **Control** |  | **P** |
| --- | --- | --- | --- | --- | --- | --- | --- | --- | --- | --- | --- | --- | --- |
|  | Name | Pre | Post | (r) | Pre | Post | (r) | Pre | Post | (r) | Pre | Post | (r) |
| **N20-P25 recovery cycle** | | |  |  |  |  |  |  |  |  |  |  |  |
|  | Index | 1.34 | 1.35 | 0.91 | 1.6 | 1.48 | 0.622 | 1.25 | 1.13 | 0.339 | 1.2 | 1.44 | 0.092 |
|  |  | [0.79, 1.55] | [0.99, 1.61] | (0.04) | [0.89, 2.21] | [1.22, 2.09] | (0.16) | [0.86, 1.61] | [0.80, 1.57] | (0.29) | [0.97, 1.46] | [1.10, 1.79] | (0.50) |
|  | 5ms | 0.9 | 0.95 | 0.85 | 0.88 | 0.89 | 0.791 | 0.66 | 0.46 | 0.97 | 0.7 | 0.82 | 0.233 |
|  |  | [0.56, 1.10] | [0.44, 1.30] | (0.07) | [0.43, 1.63] | [0.70, 1.44] | (0.09) | [0.46, 0.69] | [0.36, 0.80] | (0.02) | [0.51, 0.80] | [0.49, 1.12] | (0.36) |
|  | 20ms | 0.92 | 1.06 | 0.91 | 0.96 | 1.11 | 0.622 | 1 | 0.77 | 0.176 | 0.99 | 1.08 | 0.91 |
|  |  | [0.72, 1.40] | [0.57, 1.44] | (0.04) | [0.87, 1.49] | [0.92, 1.60] | (0.16) | [0.73, 1.35] | [0.57, 1.37] | (0.41) | [0.70, 1.18] | [0.84, 1.42] | (0.45) |
|  | 40ms | 1.09 | 1.51 | 0.47 | 1.21 | 1.42 | 0.11 | 1.01 | 1.13 | 0.91 | 1.36 | 1.32 | 0.733 |
|  |  | [0.90, 1.50] | [0.60, 1.92] | (0.23) | [0.90, 1.61] | [1.19, 1.58] | (0.48) | [0.76, 1.84] | [0.81, 1.74] | (0.45) | [1.12, 1.46] | [1.18, 1.72] | (0.11) |
|  | Thumb | 1.29 | 1.43 | 0.677 | 1.6 | 1.32 | 0.519 | 1.29 | 1.27 | 0.569 | 1.37 | 1.4 | 0.11 |
|  |  | [0.78, 2.36] | [0.77, 2.51] | (0.13) | [1.03, 2.38] | [1.11, 2.36] | (0.20) | [0.75, 1.75] | [0.76, 1.77] | (0.18) | [1.12, 1.86] | [0.96, 1.54] | (0.48) |
| **SEP** | |  |  |  |  |  |  |  |  |  |  |  |  |
|  | R5 | 0.7 | 0.61 | 0.677 | 0.65 | 0.67 | 0.266 | 0.47 | 0.54 | 0.733 | 0.58 | 0.63 | 0.97 |
|  |  | [0.55, 0.82] | [0.42, 1.15] | (0.13) | [0.44, 0.80] | [0.61, 0.78] | (0.34) | [0.39, 0.49] | [0.32, 0.60] | (0.11) | [0.51, 0.64] | [0.46, 0.65] | (0.02) |
|  | R20 | 0.93 | 0.81 | 0.85 | 0.69 | 0.76 | 0.622 | 0.84 | 0.81 | 0.569 | 0.82 | 0.8 | 0.85 |
|  |  | [0.57, 1.07] | [0.67, 0.98] | (0.07) | [0.63, 0.96] | [0.63, 0.97] | (0.19) | [0.67, 1.00] | [0.61, 1.06] | (0.18) | [0.51, 1.00] | [0.65, 1.02] | (0.07) |
|  | R40 | 1.08 | 0.95 | 0.733 | 0.79 | 0.92 | 0.092 | 0.97 | 1.07 | 0.47 | 1.08 | 1.11 | 0.47 |
|  |  | [0.72, 1.18] | [0.78, 1.26] | (0.11) | [0.70, 0.85] | [0.86, 1.16] | (0.50) | [0.77, 1.25] | [0.89, 1.17] | (0.23) | [0.88, 1.24] | [0.74, 1.22] | (0.23) |
| **SIR** | |  |  |  |  |  |  |  |  |  |  |  |  |
|  | SIRsum | 2.6 | 2.87 | 0.424 | 3.03 | 2.79 | 0.424 | 2.48 | 2.61 | 0.97 | 2.45 | 2.76 | 0.85 |
|  |  | [1.65, 3.93] | [1.93, 4.34] | (0.25) | [2.16, 4.59] | [2.26, 4.45] | (0.25) | [1.72, 3.26] | [1.64, 3.35] | (0.02) | [2.09, 3.46] | [2.44, 3.45] | (0.07) |
|  | SIRdp | 1.87 | 2.2 | **<0.001** | 1.73 | 1.89 | 0.569 | 1.2 | 1.78 | **<0.001** | 1.87 | 2.07 | 0.97 |
|  |  | [1.14, 2.53] | [1.68, 3.20] | **(0.86)** | [1.31, 3.26] | [1.29, 3.25] | (0.18) | [0.86, 1.69] | [1.37, 2.10] | **(0.86)** | [1.42, 2.19] | [1.45, 2.38] | (0.02) |
|  | SIR | 0.73 | 0.81 | **<0.001** | 0.62 | 0.64 | 0.791 | 0.51 | 0.81 | **<0.001** | 0.67 | 0.67 | 0.85 |
|  |  | [0.63, 0.77] | [0.73, 0.91] | **(0.88)** | [0.54, 0.79] | [0.57, 0.73] | (0.09) | [0.43, 0.69] | [0.64, 0.83] | **(0.86)** | [0.60, 0.73] | [0.61, 0.76] | (0.07) |

Values are median [95% confidence interval, CI], calculated by Bootstrap resample method. Random seed was set to 123 for reproducibility.

r means Rank-Biserial Correlation.

BRT = ball-rotation training, for actual execution.

Tactile = tactile control, for simple gripping.

VGI = visual-guided imagery, for motor imagery, MI.

Control for baseline conditions without intervention.

SEP = somatosensory-evoked potential.

SIR = spatial inhibition ratio.

SIRsum = the arithmetic sum of the SEP obtained by the individual stimulation of the thumb and index fingers,

SIRdp = the SEP amplitude obtained by simultaneous stimulation of the thumb and index finger.

Table S7. Statistical comparison of power spectrum

|  |  | **Value** |  | |  | | **P (r)** | |  | |  | |  |
| --- | --- | --- | --- | --- | --- | --- | --- | --- | --- | --- | --- | --- | --- |
|  | **Region** | Pre | Post | | Delta | | Pre-Post | | to Tactile | | to VGI | | to Control |
| **Gamma** | |  |  | |  | |  | |  | |  | |  |
|  | **Left Parietal** | | |  | |  | |  | |  | |  | |
|  | BRT | 0.02 | 0.03 | | 0.49 | | **0.043** | | 0.41 | | **0.028** | | **0.033** |
|  |  | [0.01, 0.02] | [0.02, 0.03] | | [0.10, 0.94] | | **(0.59)** | | (0.18) | | **(0.45)** | | **(0.44)** |
|  | Tactile | 0.02 | 0.02 | | 0.2 | | 0.85 | |  | | 0.755 | | 0.713 |
|  |  | [0.01, 0.03] | [0.02, 0.03] | | [-0.31, 0.89] | | (0.07) | |  | | (0.07) | | (0.08) |
|  | VGI | 0.03 | 0.03 | | -0.04 | | 0.622 | |  | |  | | 0.843 |
|  |  | [0.02, 0.03] | [0.02, 0.04] | | [-0.18, 0.44] | | (0.16) | |  | |  | | (0.04) |
|  | Control | 0.03 | 0.03 | | 0.18 | | 0.38 | |  | |  | |  |
|  |  | [0.02, 0.04] | [0.03, 0.04] | | [-0.06, 0.30] | | (0.27) | |  | |  | |  |
|  | **Right Parietal** | | |  | |  | |  | |  | |  | |
|  | BRT | 0.02 | 0.03 | | 0.62 | | **0.034** | | 0.242 | | **0.033** | | **0.024** |
|  |  | [0.01, 0.02] | [0.02, 0.03] | | [0.16, 0.91] | | **(0.61)** | | (0.25) | | **(0.44)** | | **(0.46)** |
|  | Tactile | 0.02 | 0.02 | | 0.15 | | 0.85 | |  | | 0.932 | | 0.755 |
|  |  | [0.01, 0.03] | [0.02, 0.03] | | [-0.44, 0.90] | | (0.07) | |  | | (0.02) | | (0.07) |
|  | VGI | 0.03 | 0.03 | | 0.04 | | 0.38 | |  | |  | | 0.843 |
|  |  | [0.02, 0.03] | [0.02, 0.04] | | [-0.16, 0.52] | | (0.27) | |  | |  | | (0.04) |
|  | Control | 0.03 | 0.03 | | 0.08 | | 0.733 | |  | |  | |  |
|  |  | [0.02, 0.04] | [0.03, 0.04] | | [-0.10, 0.27] | | (0.11) | |  | |  | |  |
| **Theta** | |  |  | |  | |  | |  | |  | |  |
|  | **Profrontal** | |  | |  | |  | |  | |  | |  |
|  | BRT | 0.08 | 0.09 | | 0.45 | | **0.034** | | 0.078 | | **0.008** | | **0.028** |
|  |  | [0.05, 0.11] | [0.08, 0.13] | | [0.21, 0.80] | | **(0.61)** | | (0.35) | | **(0.51)** | | **(0.46)** |
|  | Tactile | 0.08 | 0.08 | | 0.07 | | 1 | |  | | 0.347 | | 0.932 |
|  |  | [0.05, 0.10] | [0.06, 0.11] | | [-0.25, 0.17] | | (0.11) | |  | | (0.02) | | (0.07) |
|  | VGI | 0.09 | 0.09 | | -0.12 | | 0.519 | |  | |  | | 0.551 |
|  |  | [0.09, 0.14] | [0.07, 0.14] | | [-0.34, 0.13] | | (0.20) | |  | |  | | (0.05) |
|  | Control | 0.09 | 0.09 | | -0.06 | | 0.677 | |  | |  | |  |
|  |  | [0.07, 0.11] | [0.07, 0.10] | | [-0.12, 0.14] | | (0.13) | |  | |  | |  |
| **Delta** | |  |  | |  | |  | |  | |  | |  |
|  | **Right Parietal** | | |  | |  | |  | |  | |  | |
|  | BRT | 0.25 | 0.24 | | -0.07 | | 0.733 | | 0.59 | | 0.347 | | 0.291 |
|  |  | [0.17, 0.38] | [0.20, 0.37] | | [-0.12, 0.30] | | (0.11) | | (0.12) | | (0.20) | | (0.22) |
|  | Tactile | 0.24 | 0.2 | | -0.01 | | 0.733 | |  | | 0.41 | | 0.266 |
|  |  | [0.13, 0.37] | [0.15, 0.39] | | [-0.12, 0.19] | | (0.11) | |  | | (0.18) | | (0.24) |
|  | VGI | 0.25 | 0.29 | | 0.18 | | 0.176 | |  | |  | | **0.028** |
|  |  | [0.18, 0.31] | [0.22, 0.36] | | [-0.12, 0.54] | | (0.41) | |  | |  | | **(0.45)** |
|  | Control | 0.35 | 0.27 | | -0.11 | | 0.233 | |  | |  | |  |
|  |  | [0.27, 0.39] | [0.21, 0.35] | | [-0.30, 0.07] | | (0.36) | |  | |  | |  |

Values are median [95% confidence interval, CI], calculated by Bootstrap resample method. Random seed was set to 123 for reproducibility.

r means Rank-Biserial Correlation.

BRT = ball-rotation training, for actual execution.

Tactile = tactile control, for simple gripping.

VGI = visual-guided imagery, for motor imagery, MI.

Control for baseline conditions without intervention.

Left Parietal: including electrodes CP3, CP1, P3, P1.

Right Parietal: including electrodes CP4, CP2, P4, P2.

Prefrontal: including electrodes Fp1, Fpz, Fp2.
